# One‐Step Sixfold Cyanation of Benzothiadiazole Acceptor Units for Air‐Stable High‐Performance n‐Type Organic Field‐Effect Transistors

**DOI:** 10.1002/anie.202013625

**Published:** 2021-01-28

**Authors:** Panagiota Kafourou, Byoungwook Park, Joel Luke, Luxi Tan, Julianna Panidi, Florian Glöcklhofer, Jehan Kim, Thomas D. Anthopoulos, Ji‐Seon Kim, Kwanghee Lee, Sooncheol Kwon, Martin Heeney

**Affiliations:** ^1^ Department of Chemistry and Centre for Processable Electronics Imperial College London London W12 0BZ UK; ^2^ Heeger Center for Advanced Materials and Research Institute for Solar and Sustainable Energies Gwangju Institute of Science and Technology Gwangju Republic of Korea; ^3^ Department of Physics and Centre for Processable Electronics Imperial College London London SW7 2AZ UK; ^4^ School of Chemistry and Chemical Engineering Chongqing University Chongqing 401331 China; ^5^ Pohang Accelerator Laboratory Pohang University of Science and Technology Pohang Republic of Korea; ^6^ Division of Physical Sciences and Engineering King Abdullah University of Science and Technology (KAUST) KAUST Solar Centre Thuwal 23955-6900 Saudi Arabia

**Keywords:** field effect transistors, fluorine, nucleophilic aromatic substitution, organic electronics, semiconductors

## Abstract

Reported here is a new high electron affinity acceptor end group for organic semiconductors, 2,1,3‐benzothiadiazole‐4,5,6‐tricarbonitrile (TCNBT). An n‐type organic semiconductor with an indacenodithiophene (IDT) core and TCNBT end groups was synthesized by a sixfold nucleophilic substitution with cyanide on a fluorinated precursor, itself prepared by a direct arylation approach. This one‐step chemical modification significantly impacted the molecular properties: the fluorinated precursor, TFBT IDT, a poor ambipolar semiconductor, was converted into TCNBT IDT, a good n‐type semiconductor. The electron‐deficient end group TCNBT dramatically decreased the energy of the highest occupied and lowest unoccupied molecular orbitals (HOMO/LUMO) compared to the fluorinated analogue and improved the molecular orientation when utilized in n‐type organic field‐effect transistors (OFETs). Solution‐processed OFETs based on TCNBT IDT exhibited a charge‐carrier mobility of up to *μ*_e_≈0.15 cm^2^ V^−1^ s^−1^ with excellent ambient stability for 100 hours, highlighting the benefits of the cyanated end group and the synthetic approach.

## Graphical abstract

A new strongly electron‐accepting end group, 2,1,3‐benzothiadiazole‐4,5,6‐tricarbonitrile (TCNBT), has been prepared by a one‐step sixfold nucleophilic substitution reaction. Cyanation results in a significant enhancement of the electron affinity in comparison to the fluorinated analogue, and the material demonstrates promising n‐type performance in solution processed organic field‐effect transistors with excellent stability.

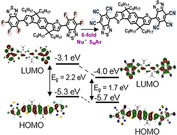

## Introduction

The development of organic semiconductors (OSCs) for use in organic electronic devices, including field‐effect transistors (OFETs), continues to attract interest because of their ease in film and device fabrication from solution. Such devices can be utilized for various applications, ranging from lightweight and flexible logic circuits to skin sensors.^[1]^ These applications typically require a good balance of charge‐carrier mobility between p‐type (hole‐transporting) and n‐type (electron‐transporting) OFETs to ensure high noise immunity and low static power consumption in the ensuing electronic circuits and systems.^[2]^ While tremendous progress has been made in the development of high‐performance p‐type OSCs for such applications,^[3]^ the performance of n‐type OSCs lags significantly behind, both in terms of charge‐carrier mobility and device stability.^[4]^

Both polymer and molecular materials have been investigated as n‐type semiconductors, but the latter have the advantage of a well‐defined molecular structure and high purity without batch to batch variations, which can improve device reproducibility.^[5]^ To facilitate electron transport, molecular OSCs should combine good π‐stacking with an electron‐deficient π‐system that facilitates electron injection and allows the lowest unoccupied molecular orbital (LUMO) energy level to be below the threshold for ambient stability in the reduced state.[^6^, ^7^, ^8^] Several promising classes of n‐type molecular materials have been developed, including those based on naphthalene diimide,[^7^, ^10^] fullerenes^[9]^ and quinoidal‐type materials.^[11]^ Often, these molecules are functionalized with fluorine and/or strong π‐acceptors such as cyano or carbonyl groups to increase their electron deficiency.[^7^, ^10^, ^11^, ^12^, ^13^] However, in many cases, the synthesis and purification of such electron‐deficient π‐systems has proven challenging, and new synthetic approaches and building blocks are required. In addition, several of the higher‐mobility materials reported either result from measurements on single‐crystal devices, which are challenging to fabricate and scale up, or have significant deviations from ideal transistor behavior, which leads to overestimated mobility values.^[14]^

A promising design motif for n‐type semiconducting materials for OFETs is the acceptor‐donor‐acceptor (A‐D‐A) architecture.[^15^, ^16^, ^17^] To date, this motif has been predominantly utilized in non‐fullerene acceptors (NFAs) for use in organic solar cells, with very few reports of their application in OFETs. Such materials are synthetically flexible, allowing the molecular packing and frontier molecular orbital energy levels to be readily tuned by varying either the electron‐rich (donor) or electron‐deficient (acceptor) part of the molecule. Most effort has focused on optimization of the donor core, and many candidates for solar applications have been identified.^[23]^ For OFETs, indacenodithiophene (IDT) and its derivatives have shown particular promise. The rigid coplanar structure of IDT enables π‐electron delocalization and reduces rotational disorder, while its four solubilizing sidechains facilitate processability.^[24]^ Less work has focused on the acceptor end groups, especially for OFET applications, and common units include indanone derivatives[^17^, ^18^] and electron‐poor heterocycles (such as benzo‐fused azoles).^[23]^

**Scheme 1:**
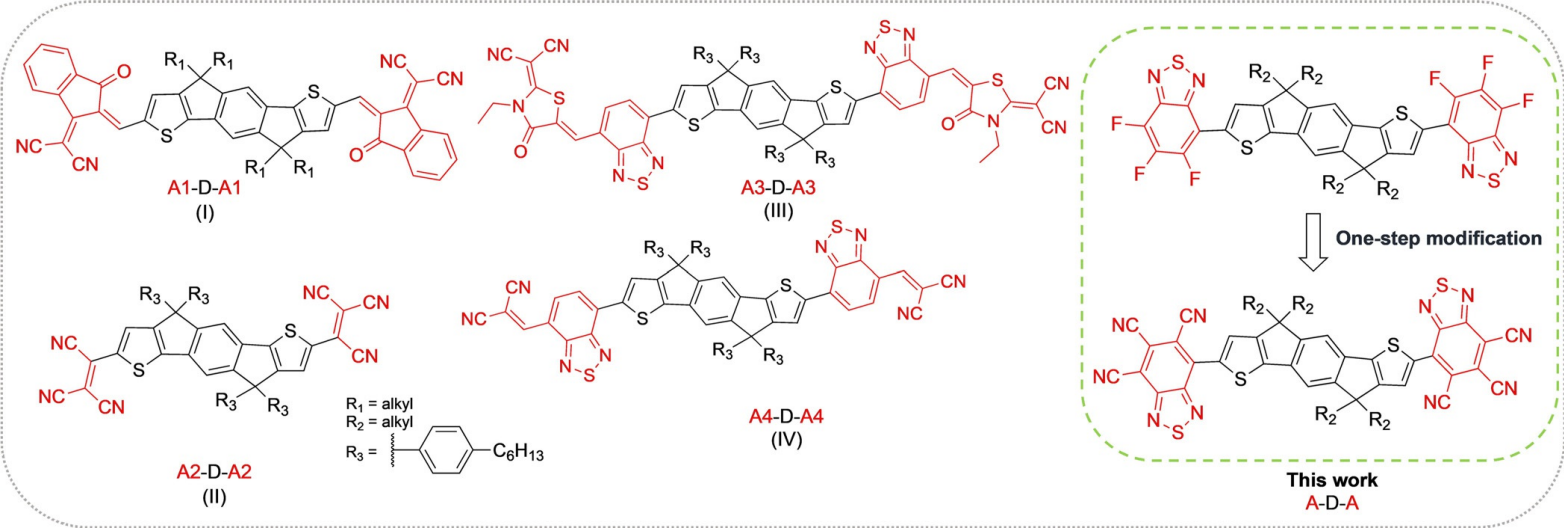
Previously reported n‐type acceptor‐donor‐acceptor (A‐D‐A) small molecules with an indacenodithiophene (IDT) core and cyanated end groups: IDT‐IC (I, R_1_=C_16_H_33_ ^[17]^; R_1_=C_6_H_13_ ^[18]^), IDT(TCV)^2^ (II),^[19]^ IDT‐RCN (III),[^20^, ^21^] IDTT‐2BM (IV);^[22]^ small‐molecule semiconductors reported in this work (right, R_2_=C_8_H_17_).

To promote stable n‐type behavior, the acceptor end group must be sufficiently strong to decrease the energy of the LUMO to less than approximately −4 eV, a value often associated with the thermodynamic stability of radical anions in air, and thus, strongly electron‐deficient acceptor end groups are required.[^8^, ^9^, ^25^] The inclusion of strongly electron‐accepting cyano (CN) groups is one approach, and examples of such cyanated acceptors in combination with IDT cores are highlighted in Scheme 1. To the best of our knowledge, only (I), showing linear mobility of *μ*_e_≈0.12 cm^2^ V^−1^ s^−1^, has been investigated in OFETs.^[17]^ However, most of the acceptor end groups of previously reported A‐D‐A molecules, including those in Scheme 1, are linked to the donor core via vinylene groups, which are known to influence the chemical stability.[^26^, ^27^, ^28^, ^29^] For example, the vinylene group of 1,1‐dicyanomethylene‐3‐indanone and its derivatives are prone to radical addition reactions, which are associated with further dimerization.^[27]^ It has also been shown to react under both basic and acidic conditions, as well as in the presence of amines.[^28^, ^29^] Furthermore, vinylene groups can undergo retro‐condensation/condensation and [2+2] cycloaddition reactions in solution, resulting in a scrambling of the end groups to form asymmetric materials.^[27]^ Developing strong electron acceptors that are directly linked to the electron‐rich core rather than through a vinylene group, can potentially solve these problems and enhance the chemical stability of these n‐type semiconductors.

Here, we focused on benzothiadiazole (BT), which is a stable electron‐deficient aromatic compound commonly used in n‐type materials, such as (III) and (IV) (Scheme 1).[^30^, ^31^] While previous work has established that the inclusion of one or two fluoro or cyano groups on BT can increase the electron affinity,^[12]^ to the best of our knowledge, trisubstituted BT derivatives have not been investigated until now. Herein, we report a convenient synthesis of 4,5,6‐trifluoro‐2,1,3‐benzothiadiazole (TFBT) and demonstrate that it can be readily attached to the IDT donor core via a one‐step C−H activation process. The resulting TFBT IDT is found to readily undergo a sixfold nucleophilic substitution of fluoride (F) by CN to afford the analogous structure with 2,1,3‐benzothiadiazole‐4,5,6‐tricarbonitrile (TCNBT) end groups.

By comparing the optoelectronic properties and structural characteristics of the TCNBT IDT semiconductor with those of its fluorinated counterpart TFBT IDT, we demonstrate that TCNBT end groups deepen both the HOMO and LUMO of the semiconductor and induce a highly ordered molecular structure, leading to good ambient stability and electron transport characteristics, respectively.

## Results and Discussion

**Scheme 2:**
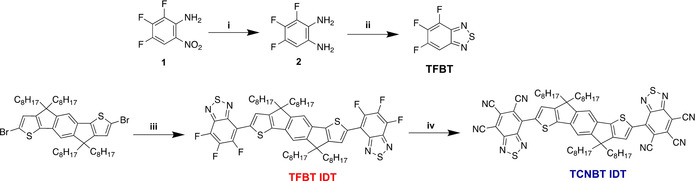
Synthesis of TFBT IDT and TCNBT IDT i) SnCl_2_⋅2 H_2_O, HCl, EtOH, 80 °C, 87 %; ii) SOCl_2_, Et_3_N, CHCl_3_, 60 °C, 70 %; iii) TFBT, Pd_2_(dba)_3_⋅CHCl_3_/P(*o*‐OMePh)_3_, PivOH, Cs_2_CO_3_, toluene, 120 °C, 38 %; iv) KCN, 18‐crown‐6, DMF, 50 °C, 65 %.

The synthesis of TFBT was based on a protocol reported previously for the synthesis of difluorinated BT (Scheme 2, top).^[31]^ Commercially available 2,3,4‐trifluoro‐6‐nitroaniline 1 was reduced with SnCl_2_ to form the corresponding diamine 2, which was subsequently reacted with thionyl chloride to form the heteroatomic ring of TFBT. Gratifyingly, we found that TFBT was well suited for cross‐coupling under direct arylation conditions, using a slight modification of the conditions reported for difluorinated BT.^[32]^ Thus, in our hands, tris(*o*‐methoxyphenyl)phosphine was found to be a suitable ligand for the reaction, and coupling with dibrominated IDT 5 afforded TFBT IDT as a red solid in moderate yields that depended on the reaction scale (from 30 to 50 %). We also investigated the reverse coupling via direct arylation of IDT itself with brominated TFBT (see Scheme S1 in the Supporting Information). Although the reaction was successful, the preparation of the required brominated TFBT was challenging. Very harsh conditions as well as long reaction times were required for this bromination. Moreover, dibrominated difluorinated BT was formed as an impurity (approx. 12 % of the product; see the Supporting Information). Following the synthesis and purification of TFBT IDT, we anticipated that direct displacement of the fluoride with cyanide by an S_N_Ar‐type reaction might be possible. To our delight, treatment with excess potassium cyanide in DMF in the presence of 18‐crown‐6 afforded TCNBT IDT by a sixfold displacement in good yields of 65 %. TCNBT IDT was isolated as a deep blue solid, and both the fluorinated and cyanated compounds were found to be highly soluble in common chlorinated organic solvents at room temperature.

**Figure 1:**
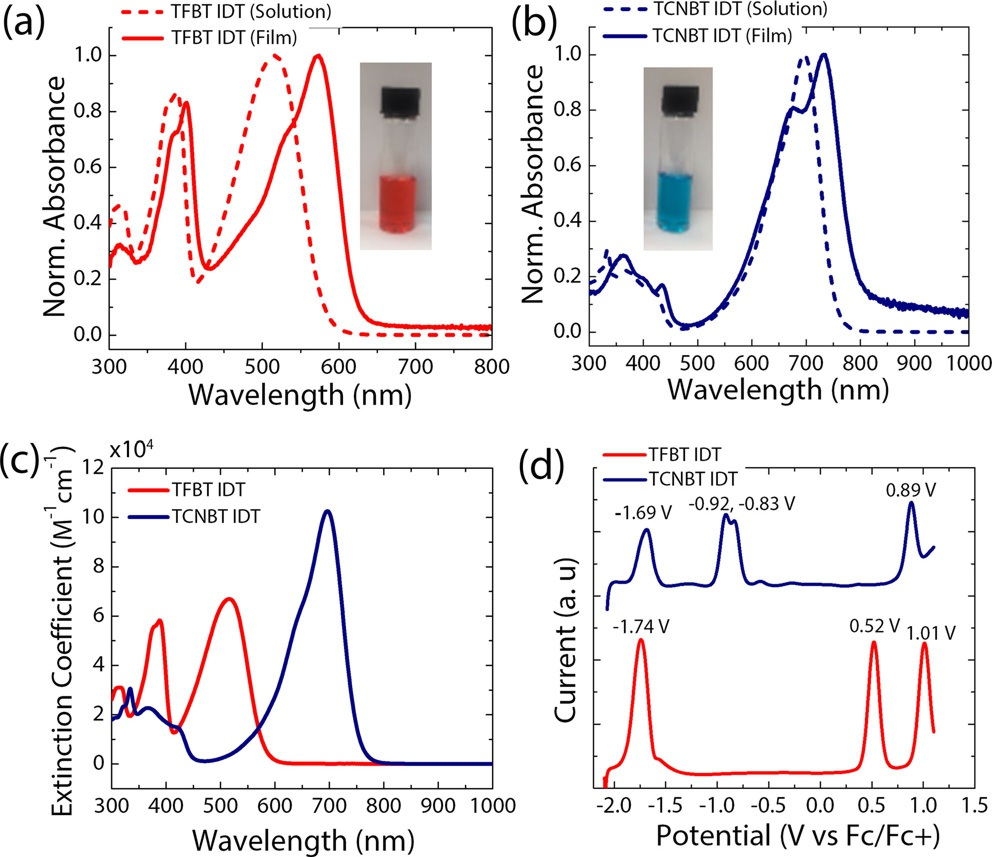
UV‐vis absorption spectra of a) TFBT IDT and b) TCNBT IDT in CHCl_3_ solution and in the solid‐state (thin‐films annealed at 80 °C). c) Extinction coefficients of TFBT IDT (red) and TCNBT IDT (blue) in CHCl_3_ (*c*=0.015 mg mL^−1^). d) SWV measurements of TFBT IDT (red) and TCNBT IDT (blue) in a 0.1 M solution of [*n*‐Bu_4_N]PF_6_ in CH_2_Cl_2_ at 25 Hz frequency, 20 mV step size, and 50 mV pulse height. The SWV potentials are referenced against ferrocene as an internal standard.^[33]^

UV/Vis absorption spectra of TFBT IDT and TCNBT IDT were recorded for samples in chloroform solution and as spun‐cast thin films annealed at 80 °C (Figure 1 a and b, respectively). TFBT IDT in solution displayed an absorption maximum at 516 nm, which was redshifted to 573 nm in the solid state. An emission peak was observed at 631 nm, corresponding to a Stokes shift of 0.20 eV in the solid‐state. The optical band gap of the TFBT IDT film, obtained from the intersection wavelength of the absorption and photoluminescence spectra, was estimated to be 2.05 eV (Figures S2 and S3). Interestingly, after substitution of the fluoride in TFBT IDT by cyanide in TCNBT IDT, the optoelectronic properties were drastically changed, with a large redshift of the absorption maximum to 696 nm in solution and a significant increase in the molar absorption coefficient (Figure 1 c). A further redshift to 733 nm was found upon thin film formation, together with the occurrence of a clear vibronic shoulder peak at approximately 678 nm, indicative of changes in the molecular packing and/or improved order upon annealing in the case of TCNBT IDT. We note that clear vibronic features appear only after annealing above 80 °C, with the absorption of the as‐cast film being broader and featureless (Figure S4). A weak emission peak was observed at 875 nm, corresponding to a slightly larger Stokes shift of 0.27 eV. The optical band gap in the solid‐state of TCNBT IDT was found to be 1.60 eV (Figure S5). The molecular orbital energy levels of both compounds were measured by square‐wave voltammetry (SWV) and cyclic voltammetry (CV) in dichloromethane solution and referenced against those of ferrocene (Table 1).

**Table 1:** Energy levels of TFBT IDT and TCNBT IDT.

	HOMO. eV (elec, SWV)^[a]^	LUMO. eV (elec, SWV)^[a]^	Eg, eV (elec)^[b]^	*λ*_max_, [nm] (sol)^[c]^	λ_min_, [nm] (film)^[c]^	Eg, [eV] (opt)^[d]^	I.P., [eV] (APS)^[e]^	LUMO, [eV] [I.P. + Eg(opt)]^[f]^
TFBT IDT	−5.3	−3.1	2.2	516	573	2.05	−5.9	−3.9
TCNBT IDT	−5.7	−4.0	1.7	696	733	1.60	−6.3	−4.7

[a] determined by SWV in CH_2_Cl_2_. [b] Determined by UV‐vis spectroscopy in CHCl_3_. [c] Determined by UV‐vis spectroscopy of thin films annealed at 80 °C. [d] Determined from the intersection wavelength of the absorption and photoluminescence spectra in the solid‐state. [e] Determined by ambient photoemission spectroscopy (APS) on thin films annealed at 80 °C, error ±0.05 eV. [f] Estimated by adding Eg(opt) to the ionization potentials determined by APS.

The CV results demonstrate that both materials exhibit multiple quasi‐reversible oxidation and reduction peaks in solution (Figure S6). The oxidation and reduction potentials were more readily extracted from the SWV measurements (Figure 1 d), with the HOMO and LUMO energy levels of TFBT IDT estimated to be at −5.3 eV and −3.1 eV, respectively. A second oxidation peak is also observed at higher potential, but only a single reduction peak is seen in the electrochemical window investigated.

Cyanation has a significant impact on the electrochemistry, with a decrease in the HOMO energy by 0.4 eV and the LUMO energy by 0.9 eV (Figure 1 d), indicating a significant increase in electron affinity compared to its fluorinated counterpart. In addition, multiple reversible reduction peaks were observed, possibly corresponding to single‐electron reduction of each TCNBT end group, followed by further reduction. The measurement results were in reasonable agreement with the values obtained via density functional theory (DFT) calculations at the B3LYP level of theory with the 6‐31G(d,p) basis set, which also showed that cyanation resulted in a larger downshift of the LUMO energy compared to the HOMO energy, with HOMO/LUMO energy levels of −5.1/−2.9 eV for TFBT IDT and −6.1/−4.1 eV for TCNBT IDT (Figure S7, S8). For both materials, we were unable to perform CV in the solid state due to partial dissolution of the film during measurement, so the ionization potential in the solid state was determined by ambient photoemission spectroscopy (APS).

A trend similar to that observed by SWV was seen for the annealed films, with the APS‐obtained HOMO energies of TFBT IDT and TCNBT IDT being −5.9 eV and −6.3 eV, respectively (Figures S9 and S10). Compared to the value determined from the SWV measurement, the lower absolute HOMO values determined from the APS measurement may be due to the differences between the solution and solid‐state samples or due to known discrepancies between electrochemistry and photoelectron techniques.^[34]^ Finally, the solid‐state LUMO energies were estimated by adding the optical band gap (Table 1). By comparing the optoelectronic properties of both compounds, it is evident that the substitution of the fluorides by cyanides results in a downshift of both the HOMO and LUMO energy levels, but the effect is much larger on the LUMO, resulting in a narrowing of the band gap and much higher electron affinity. The Fermi levels, which were measured by the Kelvin probe, also indicated that TCNBT IDT has a dominant n‐type character, with the work function being close to the LUMO energy level (Figure S10). In contrast, the Fermi level of TFBT IDT was found to be in the middle of the band gap denotative of more intrinsic semiconductor (Figure S9).

The stability of both materials was investigated under various stimuli. Thermogravimetric analysis (TGA) demonstrated that both materials exhibited excellent thermal stability, with an onset of decomposition under dry air of 363 °C and 375 °C for TFBT IDT and TCNBT IDT, respectively (Figure S11). Differential scanning calorimetry (DSC) measurements also showed that both materials exhibited good stability under repeated thermal cycling, with little change observed in the position and enthalpy of the melting peak (Figure S12). Interestingly, a large difference in the melting point was observed, despite the small difference in molecular mass between the materials, with TFBT IDT melting at ≈140 °C and TCNBT IDT melting at ≈240 °C, suggestive of significant differences in their intra and intermolecular interactions.

As discussed earlier, the chemical instability of vinylene‐linked end groups in the presence of basic amines has been noted.[^28^, ^29^] Modifiers such as polyethylenimine (PEI) are often used to decrease the work function of metal electrodes to enhance electron injection, and thus stability in the presence of amines is potentially important.^[35]^ When solutions of TCNBT IDT were treated with linear polyethylenimine, no color change was observed, whereas under identical conditions, the vinylene‐based acceptor ITIC^[36]^ showed a color change and loss of the lowest energy peak in the UV‐vis absorption spectrum (Figure S13), in agreement with literature reports.^[28]^

**Figure 2:**
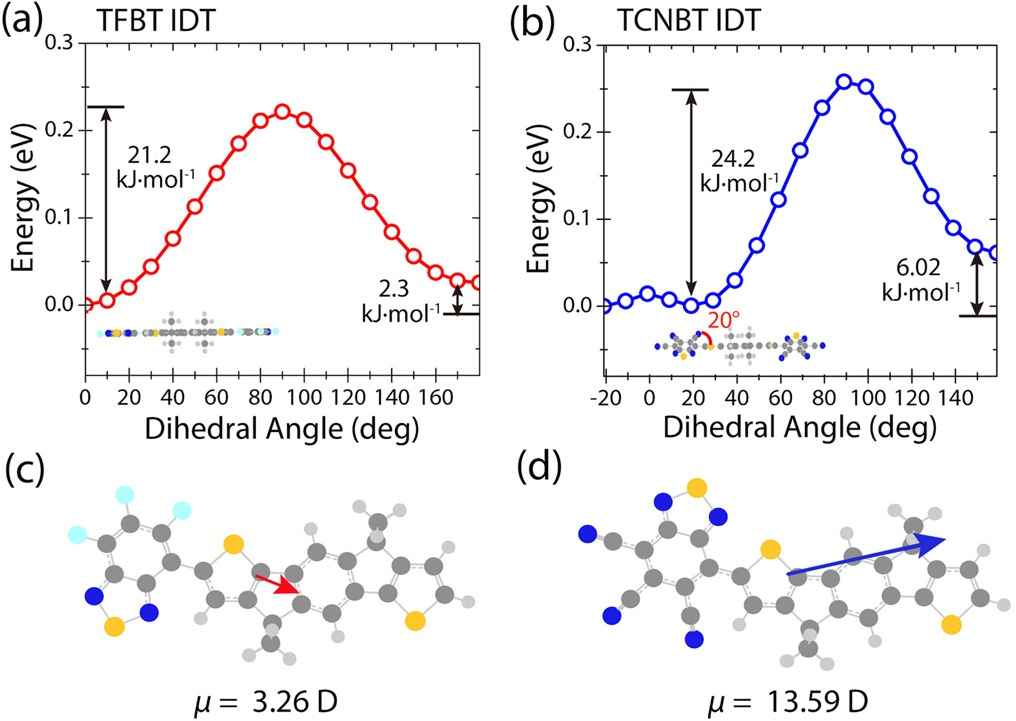
DFT‐derived potential energy for the torsional degree of freedom between 0 and 180 degrees in steps of 10° for directions of the inner thiophene rings next to the BT unit. a) Potential energy scan of the TFBT IDT dihedral angle. The structure is planar when optimized. b) Potential energy scan of the TCNBT IDT dihedral angle. There is a 20° twist between TCNBT and IDT at the minimum energy conformation; Calculation of the dipole strength between c) TFBT—IDT and d) TCNBT—IDT, for the minimum energy conformers.

To compare the molecular conformation of TFBT IDT and TCNBT IDT, we calculated the minimum energy structures and the dipole strength between the core and end groups by DFT simulations, using the B3LYP functional and a 6.31g(d,p) basis‐set (Figure 2).

Comparing the dipole strength between the fluorinated and cyanated end groups it is evident that the electron withdrawing effect of the TCNBT end group is much stronger than the effect of the TFBT end group, by approximately 10 Debye (Figure 2 c,d). Additionally, molecular optimization results suggest that the two materials adopt different relative orientations of the IDT and the BT end group, with an *anti* conformation (with respect to the sulfur atoms in IDT and BT) favored for TFBT IDT, whilst TCNBT IDT prefers a *syn* conformation. In addition, TFBT IDT is predicted to have a completely planar backbone, whereas TCNBT IDT has a slight twist (20°) between the IDT core and the TCNBT end group. These results were further investigated by potential energy scans, in which the angle between the end group and the IDT was systematically changed between 0 and 180 degrees (Figure 2 a,b).

These results confirmed that TFBT IDT had an energy minimum in the *anti* geometry, with the *syn* geometry higher in energy by 2.3 kJ mol^−1^. In the case of TCNBT IDT, the difference between the favored *syn* confirmation and the *anti* is a more significant 6.0 kJ mol^−1^. In addition, although the energy minimum at a twist of 20° was confirmed, the energetic penalty for planarization (i.e. 0°) is modest (<0.1 kJ mol^−1^) and easily overcome in the solid‐state.

**Figure 3:**
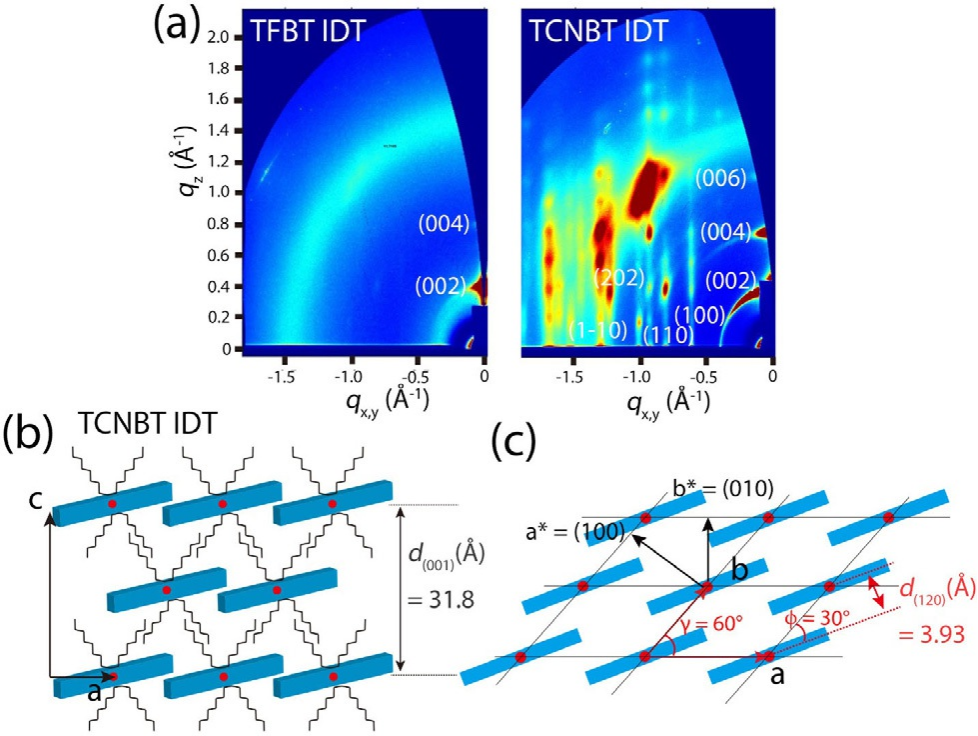
a) 2D GIWAXS patterns of TFBT IDT (left) and TCNBT IDT (right). b) Out‐of‐plane and c) in‐plane direction of TCNBT IDT in the solid‐state. Red dots indicate the center position of the TCNBT IDT molecule.

To understand the structural evolution of TFBT IDT and TCNBT IDT, we performed grazing‐incidence wide‐angle X‐ray scattering (GIWAXS) measurements on the corresponding thin films (see Methods for further details). Note here that we found that there was no structural change in TFBT IDT with temperature, while TCNBT IDT gave rise to many intense diffraction peaks as the annealing temperature increased to 150 °C (Figure S14). Hence, we chose the GIWAXS patterns for the TCNBT IDT film annealed at 150 °C to define the crystal structure more clearly. Figure 3 a shows two‐dimensional GIWAXS patterns of the TFBT IDT and TCNBT IDT films. For the TFBT IDT film, weak diffraction peaks were observed along the out‐of‐plane direction at *q*_z_ (Å^−1^)=0.401 for the (002) plane and at *q*_z_ (Å^−1^)=0.802 for the (004) plane, which were associated with a lamellar spacing of the plane (2π/0.2005=31.3 Å). The diffusive amorphous ring pattern may be attributed to poor molecular orientation, possibly as a result of the weak molecular dipole,^[37]^ which may limit lateral charge transport across the film. However, for the TCNBT IDT film, a series of high‐intensity diffraction peaks were observed at *q_z_* (Å^−1^)=0.398, 0.796, and 1.19 for the (002) (004) and (006) planes along the *q*_z_ axis. In addition, much clearer (100), (11*l*) and (01*l*) diffraction families of reflections were observed at *q*_*x*,*y*_ (Å^−1^)=0.62, 0.81, and 0.93, respectively. Further structural analysis based on the diffraction peaks revealed that the crystal structure of the TCNBT IDT film conforms to a monoclinic unit cell with lattice parameters *a*=11.7165 Å, *b*=7.8505 Å, *c*=31.8297 Å, *α*=90°, *β*=90° and *γ*=60° (Figure S15–S17).

Based on this analysis, we propose a lamellar stacking and π‐π stacking structure of TCNBT IDT along the out‐of‐plane and in‐plane directions, respectively (Figure 3 b and c). For the out‐of‐plane direction, the molecules are vertically stacked in identical rows shifted with respect to each other; the molecules within each row partially overlap along the row direction, presumably due to the strong interaction between an acceptor TCNBT unit of one and a donor IDT unit of the other, in agreement with the strong molecular dipole. This arrangement is analogous to a brick‐wall structure. Similarly, along the in‐plane direction, the molecules are arranged in rows with a brick‐wall structure, where the molecules are slightly tilted (*φ*=30°) to partially overlap due to strong intermolecular interactions.

From the proposed molecular packing structure, the π‐π stacking distance was determined to be *d*(120) (Å)=3.93. Compared to TFBT IDT, the highly ordered molecular packing with the brick‐wall structure and *γ*=60° of TCNBT IDT might be interpreted as suggesting a significant change in intermolecular interaction between adjacent molecules. Thus, it would be reasonable to conclude that the strong TCNBT‐IDT intermolecular interaction in TCNBT IDT molecules promotes a stable crystal structure upon film formation and an overall enhancement of in‐plane π–π stacking for charge transport.

To understand the effect of annealing on the molecular conformation, the Raman spectra of TCNBT IDT at different annealing temperatures were obtained and compared to DFT simulated Raman spectra (Figure S18). The simulated and experimental spectra align reasonably well; a full vibrational mode peak assignment can be found in the SI. Upon annealing at 80 °C, there are clear peak shifts and intensity changes in the experimental Raman spectra. Interestingly, if we simulate the vibrational spectra at different molecular conformations, namely, disrupting planarity by rotating about the IDT‐BT dihedral, we can reproduce many of the experimentally observed peak changes. Therefore, upon annealing, it appears that the TCNBT IDT molecules become more planar, which correlates well with the improved crystallinity observed in the annealed films. The simulated HOMO energy levels deepen as the planarity of TCNBT IDT is increased (Figure S19); similarly, there is a deepening in the solid‐state TCNBT IDT HOMO energy level as measured by APS from −6.1 in as‐cast films to −6.3 eV in 80 °C annealed films (Figure S10), further corroborating the increase in planarity observed in the Raman spectra. As the molecule becomes more planar the HOMO energy level is delocalized more across both the IDT and TCNBT units; this increased delocalization and larger contribution from the electron‐withdrawing groups stabilizes the HOMO energy level. As with the absorption spectra, both the Raman spectra and HOMO level do not change further if annealing occurs at higher temperatures.

The charge‐transport properties of TFBT IDT and TCNBT IDT were investigated using an OFET device configuration. Initial studies on TFBT IDT were performed in a bottom contact/top gate (BC/TG) architecture using Au electrodes and Cytop as the gate dielectric. In agreement with the energetic measurements and structural analysis above, TFBT IDT showed ambipolar OFET performance, with a moderate electron mobility of *μ*_e_≈1.5×10^−2^ cm^2^ V^−1^ s^−1^ and a noticeably lower hole mobility of *μ*_h_≈2.4×10^−3^ cm^2^ V^−1^ s^−1^ (Figure S20). In contrast, the corresponding OFET with TCNBT IDT annealed at 150 °C exhibited purely n‐type charge transport, with promising linear and saturation electron mobilities of *μ*_e,max_≈0.04 and 0.11 cm^2^ V^−1^ s^−1^, respectively (Figure S21 and Table S2). Thus, all further investigations were performed on TCNBT IDT.

**Figure 4:**
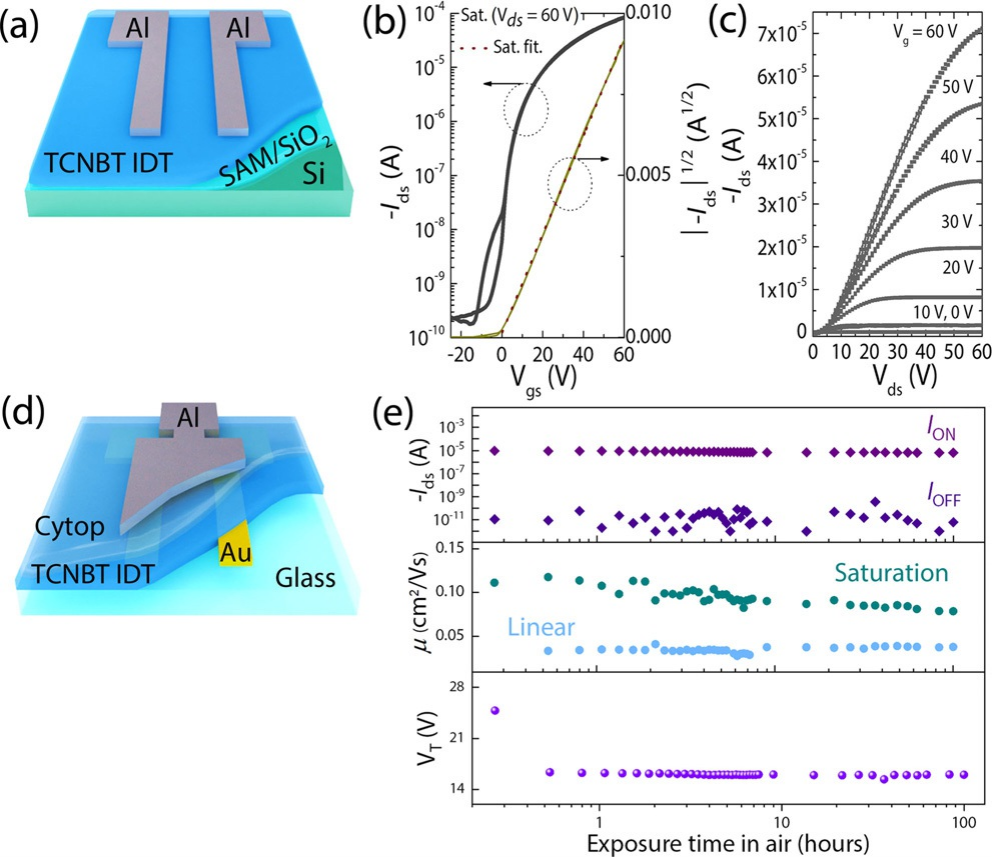
a) TC/BG OFET architecture, W L^−1^ 1500/50 μm. b) Transfer and c) output curves for optimized TCNBT IDT devices. d) BC/TG OFET architecture, W L^−1^ 1000/30 μm, for TCNBT IDT device stability test. e) Ambient device stability results.

The influence of thermal annealing on device performance was investigated in a more convenient top contact, bottom gate (TC/BG) device, in which the semiconductor was annealed before deposition of the S/D Al electrodes (Figure 4 a). Aluminum electrodes were used because of their improved performance over gold (Figure S22) in this device geometry. A significant improvement in device performance was observed upon annealing the as‐cast film, with an optimal annealing temperature of 150 °C found to improve charge‐carrier mobility (Figure S23), as evident by the significant increase in the channel current by approximately 64 %. A second brief annealing step after the deposition of the electrode further improved the performance (Figure S24), and the optimal device measurements are shown in Figure 4 b and c. The devices exhibited a saturated mobility of *μ*_e,max_≈0.15 cm^2^ V^−1^ s^−1^, with a threshold voltage *V*_T_=0.1 V and an ON/OFF current ratio of ≈5×10^5^ (Table S2). Notably, the square root plot of the drain current was linear, with no evidence of device non‐idealities apparent in some high‐mobility devices.^[36]^

To probe the device stability, the annealed films were stored under ambient conditions and were periodically tested every three weeks over a period of 10 weeks (Figure S25). Since the aluminum source/drain electrodes rapidly oxidized under these conditions, fresh contacts were deposited on top of the active layer before testing. The results demonstrated that the unannealed devices exhibited some deterioration in device performance but that the annealed semiconductor devices appeared stable with little change in performance. Changing back to a BC/TG device using gold electrodes enabled the stability of the whole device to be probed (Figure 4 d). The device was measured in air for 100 hours and retained its good performance (Figure 4 e). Promisingly, both linear and saturation electron mobility remained almost constant throughout the measurement, with values of *μ*_e_≈0.04 and 0.11 cm^2^ V^−1^ s^−1^, respectively. The ON/OFF current ratio remained stable, as did the threshold voltage (after an initial drop to 15 V when exposing the device to air). We believe that the good stability under ambient conditions relates to the very low‐lying LUMO level of TCNBT IDT, which improves the thermodynamic stability of the reduced species against moisture and oxygen. Furthermore, an increase in planarity and further deepening of the LUMO levels contribute to the improved stability after annealing (Figure S10), as we have previously shown that molecular planarity and conformational locking can improve the stability of IDT‐BT‐containing molecules.^[38]^

## Conclusion

In conclusion, we demonstrate a high‐performance acceptor‐donor‐acceptor (A‐D‐A) n‐type OSC based on a novel 2,1,3‐benzothiadiazole‐4,5,6‐tricarbonitrile (TCNBT) acceptor. An efficient synthetic method is reported based on a one‐step sixfold nucleophilic substitution of a fluoride containing precursor with cyanide. The replacement of fluorine with cyano groups is found to have a significant impact on the electronic properties, decreasing the HOMO and LUMO energy levels by 0.4 eV and 0.9 eV with respect to the fluorinated analogue. The resulting TCNBT IDT exhibits a stable monoclinic crystal structure upon film formation, with annealing found to improve molecular planarity and enhanced in‐plane π–π stacking for charge transport, as confirmed by advanced 2D GIWAXS measurements. Consequently, we succeeded in fabricating stable n‐type OFET devices with a charge‐carrier mobility of up to *μ*_e_≈0.15 cm^2^ V^−1^ s^−1^ and a prolonged device lifetime of more than 100 hours in ambient atmosphere. We believe these results demonstrate that TCNBT is a promising high electron affinity acceptor end group with good chemical stability, which will be of interest in the development of future materials for organic electronic applications.

## Conflict of interest

The authors declare no conflict of interest.

## Supporting information

As a service to our authors and readers, this journal provides supporting information supplied by the authors. Such materials are peer reviewed and may be re‐organized for online delivery, but are not copy‐edited or typeset. Technical support issues arising from supporting information (other than missing files) should be addressed to the authors.

Supplementary
