# Supplementary material for: One‐Step Sixfold Cyanation of Benzothiadiazole Acceptor Units for Air‐Stable High‐Performance n‐Type Organic Field‐Effect Transistors
Source: Angew Chem Int Ed Engl. 2021 Jan 28;60(11):5970–7. doi: 10.1002/anie.202013625 (PMC7986693; doi:10.1002/anie.202013625)
Supplement: Supplementary file 1 — Supplementary [file ANIE-60-5970-s001.pdf]

Angewandte Chemie
Eine Zeitschrift der Gesellschaft Deutscher Chemiker

Supporting Information

One-Step Sixfold Cyanation of Benzothiadiazole Acceptor Units for Air-Stable High-Performance n-Type Organic Field-Effect Transistors

Panagiota Kafourou, Byoungwook Park, Joel Luke, Luxi Tan, Julianna Panidi, Florian Glöcklhofer, Jehan Kim, Thomas D. Anthopoulos, Ji-Seon Kim, Kwanghee Lee, Sooncheol Kwon, and Martin Heeney**

anie_202013625_sm_miscellaneous_information.pdf

Supporting Information

Table of Contents

1. Methods 2
2. Synthesis 4
3. Optoelectronic characterization of TFBT IDT and TCNBT IDT 8
4. Characterization of the energy level of TFBT IDT and TCNBT IDT 10
5. Thermogravimetric analysis (TGA) 12
6. Differential scanning calorimetry (DSC) 13
7. Stability test for TCNBT IDT 13
8. Structural characterization of TCNBT IDT 14
9. Raman-structure changes upon film annealing 17
10. Transistor performance and optimization 18
11. TCNBT IDT stability tests 21
12. NMR spectra 22

1. Methods

Material synthesis

2,3,4-Trifluoro-6-nitroaniline **1** was purchased from Fluorochem. Pd_2dba_3 catalyst was purchased from Sigma-Aldrich and was recrystallised following the procedure of Zalesskiy and Ananikov,[1] forming the adduct $Pd_2dba_3{\cdot}CHCl_3$. Further purification of **TFBT IDT** and **TCNBT IDT** was conducted using recycling GPC from Japan Analytical Industry Co, Ltd; the eluent used was chloroform. The system consisted of a high pressure liquid chromatography apparatus (JAI LaboACE LC 5060 series) equipped with a pump (P-LA60, flow rate 10 ml min−1), a UV detector (UV-VIS4ch LA, λ = 210 nm, 254 nm, 330 nm, 400 nm) and two columns (Jaigel 2HR and 2.5HR, inner diameter 20 mm × length 600 mm each). Nuclear magnetic resonance (NMR) spectra were recorded on Bruker AV-400 (400 MHz) spectrometers in $CDCl_3$ using the residual solvent resonance of *o*-DCB-d_4, or D_2-*1,1,2,2*-tetrachloroethane. UV-Vis spectra in chloroform solution were recorded in a UV-1601 Shimadzu UV-vis spectrometer.

Material characterizations

Cyclic and square-wave voltammograms were recorded using a Metrohm Autolab PGStat101 potentiostat/galvanostat. The experimental setup consisted of an Ag/Ag^+ reference electrode, a platinum wire counter electrode and an FTO working electrode, and all measurements were carried out under nitrogen at room temperature. Measurements were performed in anhydrous, degassed solutions of CH_2Cl_2 with tetrabutylammonium hexafluorophosphate (0.1 M) electrolyte. After each measurement, an arbitrary amount of ferrocene was added to the solution as an internal reference. Square-wave voltammetry (SWV) measurements were conducted in a 0.1 M solution of [*n*-Bu_4N]PF_6 in CH_2Cl_2 at 25 Hz frequency, 20 mV step size, and 50 mV pulse height. The SWV potentials were referenced to those of ferrocene when a ferrocene/ferrocenium reference redox system of 4.8 eV below the vacuum level was used as an internal standard, and the conversion from electrochemical potentials to electron volts was done using the formula $E(eV) = -E_{redox} - 4.8$ eV.[2, 3] Any solvent effects were neglected.

Raman spectroscopy

A Renishaw inVia Raman microscope with a 50× objective in a backscattering configuration was used to collect both PL and Raman spectra. Films for the measurements were prepared by spin-coating from 10 mg mL^{-1} solutions in chloroform onto glass substrates. To avoid photooxidation all samples were measured in a nitrogen purged Linkam sample chamber. All

measurements were taken with a defocused laser spot with a radius of ≈ 10 μm, laser powers and acquisition times were optimised and kept consistent between samples. Both Raman and PL spectra were recorded using 514 nm laser excitation. Raman spectra were baselined by subtraction of a polynomial function; PL spectra are shown with no baselining or instrument response corrections.

Ambient Photoemission Spectroscopy (APS)

Ionisation potentials were measured by ambient photoemission spectroscopy (APS) measurements using an APS04 air photoemission system (APS04, by KP Technology) and a 2 mm gold tip under atmospheric conditions. Films for the measurements were prepared by spin-coating from 10 mg mL^{-1} solutions in chloroform onto ITO substrates. Films were annealed at 80°C, 150°C and 200°C for three minutes in an inert atmosphere. All samples were grounded via the ITO substrate. Measurements were taken at multiple positions on the films to ensure reproducibility. The APS data were processed using the protocol described by Baikie et al.[4] This involved taking the cube root of the measured photoemission, fitting the resultant linear region, and extrapolating to zero photoemission to find the HOMO level of the semiconductor.

Density functional theory (DFT) calculation

DFT calculations were conducted using Gaussian 09 software on the Imperial College High-Performance Computing Service.[5] All simulations were carried out on single molecules in the gas phase at the B3LYP level of theory with the basis set 6-31G(d,p).[6-8] Alkyl side chains were replaced by methyl groups to reduce the computation time. Structures were optimized to a local minimum energy conformation, and frozen dihedral angles were used to simulate molecular conformational changes. Frequency calculations were carried out to simulate the Raman spectra; an empirical scaling factor of 0.97 was applied to the calculated wavenumber.[9] Visualization of the simulated vibrational modes using GaussView 6.0.16 software was used to aid Raman peak assignment alongside consultation with the literature.

Device fabrication and characterizations

Transistor characterization was carried out under nitrogen using a Keithley 4200 parameter analyser. All films were prepared and characterized under inert atmosphere. Bottom gate/top contact (BG/TC) devices were fabricated on heavily doped n^+-Si(100) wafers with 300 nm thick thermally grown SiO_2. The Si/SiO_2 substrates were treated with trichloro(octadecyl)silane (ODTS) to form a self-assembled monolayer. **TCNBT IDT** was

dissolved in chloroform (20 mg mL^{-1}) and spun cast at 2000 rpm for 60 seconds from a room temperature solution and annealed at 150°C for 3 minutes. Al (50 nm) or Au (30/40 nm) source and drain electrodes were deposited under vacuum through shadow masks. The channel width and length of the transistors were 1500 μm and 50 μm, respectively. The transfer and output characteristics were determined in a N_2-filled glove box using a Keithley 4200 source meter. The saturation-regime mobility of the transistor was determined using the equation $I_{ds} = (WC_i/2L)\mu_{sat}(V_g-V_T)^2$, where I_{ds} is the source–drain current, C_i (10 nF cm^{-2}) is the capacitance per unit area, L is the channel length, W is the channel width, and V_g and V_T are the gate and threshold voltages, respectively.

Top gate/bottom contact (TG/BC) devices were fabricated on glass substrates using Au (40 nm) source–drain electrodes and a Cytop dielectric. The channel width and length of the transistors were 1000 μm and 30/40 μm, respectively. Both **TFBT IDT** and **TCNBT IDT** were dissolved in chloroform (10 mg mL^{-1}) and spin coated at 2000 rpm from a room temperature solution for 60 seconds before being annealed at 120°C and 150°C for 3 minutes, respectively. The saturation-regime mobility of the transistor was determined using the equation $I_{ds} = (WC_i/2L)\mu_{sat}(V_g-V_T)^2$, where I_{ds} is the source–drain current, C_i (2.1 nF cm^{-2}) is the capacitance per unit area, L is the channel length, W is the channel width, and V_g and V_T are the gate and threshold voltages, respectively. For the air stability TFT measurements, the saturation mobility was calculated from the slope of the second derivative of the drain current versus gate voltage.

2. Synthesis

3,4,5-Trifluorobenzene-1,2-diamine (2). To a solution of 2,3,4- trifluoro-6-nitroaniline **1** (10 g, 52.1 mmol) in absolute ethanol (300 mL) was added dropwise concentrated HCl (170 mL). Once compound **1** was dissolved, $SnCl_2 \cdot 2H_2O$ (82 g, 365 mmol) was added in several portions. The reaction mixture was heated to 80 to 85°C for 6 hours. After cooling down to room temperature, the solution was decanted in 500 mL of ice water and the solution was neutralised with 50% NaOH to reach a pH of 7-8. After filtration, the filtrate was extracted with ethyl acetate (3 x 500 mL) and dried over $MgSO_4$. Evaporation of ethyl acetate under reduced pressure afforded the product as brown solid (7.33 g, 45.2 mmol, 87%); m.p. 75-78°C; *^1H NMR (400 MHz, $CDCl_3$):* δ = 6.35 (ddd, J = 12, 6.9, 2.3 Hz, 1H), 3.47 (s, 2H), 3.22 (s, 2H) ppm; *^{19}F-{H} NMR (376 MHz, $CDCl_3$):* δ = -148.0 (dd, J = 22, 2.5 Hz), -154.4 (dd, J = 22, 2.5 Hz),

-172.9 (td, *J* = 24, 2.8 Hz) ppm; *HRMS* (M+, EI): *m/z* calcd. for $C_6H_5F_3N_2$ 162.0422, found 162. 0427.

4,5,6-Trifluoro-2,1,3-benzothiadiazole (TFBT). To a solution of 3,4,5-trifluorobenzene-1,2-diamine **2** (7.33 g, 45.2 mmol) in chloroform (~150 mL) and triethylamine (18.3g, 181 mmol), was added thionyl chloride (10.8 g, 90.4 mmol) at 0°C dropwise. After addition, the mixture was refluxed for 12 hours, and allowed to cool to room temperature. After the addition of 150 mL of water the solution was extracted with CH_2Cl_2 (2 x 100 mL). The combined organics were further washed with water (100 mL) and brine (100 mL) and finally dried over $MgSO_4$. The solvent removed under reduced pressure and the crude product was purified by column chromatography [eluent: hexane/ CH_2Cl_2, 1:1 (v:v)] to afford an off-white cotton/flake type solid (6.05 g, 31.8 mmol, 70%); m.p. 28-30°C; *^1H NMR (400 MHz, $CDCl_3$): δ* = 7. 61 (ddd, *J* = 15.8, 6.6, 2.3 Hz, 1H) ppm; *^{19}F-{H} NMR (376 MHz, $CDCl_3$): δ* = -126.1 (dd, *J* = 18, 5.3 Hz), -142.5 (dd, *J* = 18, 5.3 Hz), -154.3 (dd, *J* = 17, 15 Hz) ppm; FTIR (ATR, cm^{-1}): 3084 (w, aromatic CH), 1653, 1559, 1473, 1355, 1304, 1178, 1080, 975, 856, 782 (all s); HRMS (M+, EI): *m/z* calcd. for $C_6HF_3N_2S$ 189.9848, found 189.9856.

4,9-Dihydro-s-indaceno[1,2-b:5,6-b']bisthiophene-2,7-diyl)bis(5,6,7-trifluoro-2,1,3-benzothiadiazole) (TFBT IDT). Compound **5** (2.0 g, 2.30 mmol), **TFBT** (1.3 g, 6.90 mmol), $Pd_2(dba)_3 \cdot CHCl_3$ (119 mg, 5%) , tris(*o*-anisyl) phosphine (70 mg, 0.23 mmol), pivalic acid (70 mg, 0.69 mmol) and caesium carbonate (2.4 g, 6.90 mmol) were added in a sealed vial and purged with nitrogen. Degassed, anhydrous toluene (15 mL) was added and the mixture was heated to 120°C for 12h. After cooling, toluene was removed under reduced pressure and the residue was dissolved in CH_2Cl_2. The organic phase was washed with water and brine and the crude product was purified using column chromatography [eluent: hexane/ CH_2Cl_2 3:1 (v:v)] and further purified using recycling GPC in chloroform. The product was isolated as deep red solid (0.95 g, 0.87 mmol, 38%); m.p. (DSC) 148-149°C; *^1H NMR (400 MHz, $CDCl_3$):* δ = 8.14 (s, 2H), 7.44 (s, 2H), 2.14-1.92 (m, 8H), 1.12-0.90 (m, 48H), 0.78 (t, *J* = 7.1 Hz, 12 H*) ppm; ^{19}F-{H}- NMR (376 MHz, $CDCl_3$): δ* = -127.2 (d, *J* = 16 Hz), -146.5 (d, *J* = 16 Hz), -153.3 (t, *J* = 16 Hz) ppm; UV/Vis ($CHCl_3$): λ_{max} (ε): 315 nm (31268 M^{-1} cm^{-1}), 388 nm (58475), 516 nm (66980); *MS (MALDI-TOF)*: isotopic cluster at *m/z* 1091 [M^+].

7,7'-(4,4,9,9-Tetramethyl-4,9-dihydro-s-indaceno[1,2-b:5,6-b']bisthiophene--2,7-diyl)di(2,1,3-benzothiadiazole-4,5,6-tricarbonitrile) (TCNBT IDT). TFBT IDT (400 mg, 0.37 mmol), KCN (167 mg, 2.6 mmol) and 18-crown-6 (9.8 mg, 0.037 mmol) were added to a sealed vial and purged with nitrogen. Degassed, anhydrous DMF (10 mL) was added and the mixture heated at 50°C for 12 h. After cooling, the reaction mixture was decanted in 100 mL

of water and the mixture was extracted with CH_2Cl_2 (3 x 100 mL). Note: The aqueous extracts were treated with ammonia solution (28%) to destroy any residual cyanide present. The combined organic layers were dried over Na_2SO_4 and CH_2Cl_2 was evaporated under reduced pressure. The crude product was purified by column chromatography over silica [eluent: hexane/ CH_2Cl_2 1:4 (v:v)] and further purified by recycling GPC in chloroform. The product was isolated as deep blue solid (270 mg, 0.24 mmol, 65%); m.p. (DSC) 248-249°C; *^1H NMR (400 MHz, $CDCl_3$):* δ = 8.58 (s, 2H), 7.58 (s, 2H), 2.18-1.94 (m, 8H), 1.18-0.93 (m, 48H), 0.78 (t, J = 7.1 Hz, 12H) ppm; *^{13}C NMR (100 MHz, $CDCl_3$):* δ = 157.7, 155.9, 154.4, 152.9, 152.0, 138.0, 136.9, 135.6, 129.8, 122.3, 115.8, 115.4, 112.8, 111.8, 107.2, 105.6, 54.7, 38.8, 31.6, 29.8, 29.2, 29.1, 24.4, 22.5, 13.9 ppm; FTIR (ATR, cm^{-1}): $\tilde{\nu}$ = 2427 (s, -CN); UV/Vis ($CHCl_3$): λ_{max} (ε): 696 nm (102692 M^{-1} cm^{-1}); *MS (MALDI-TOF)*: isotopic cluster at *m/z* 1133 [M^+].

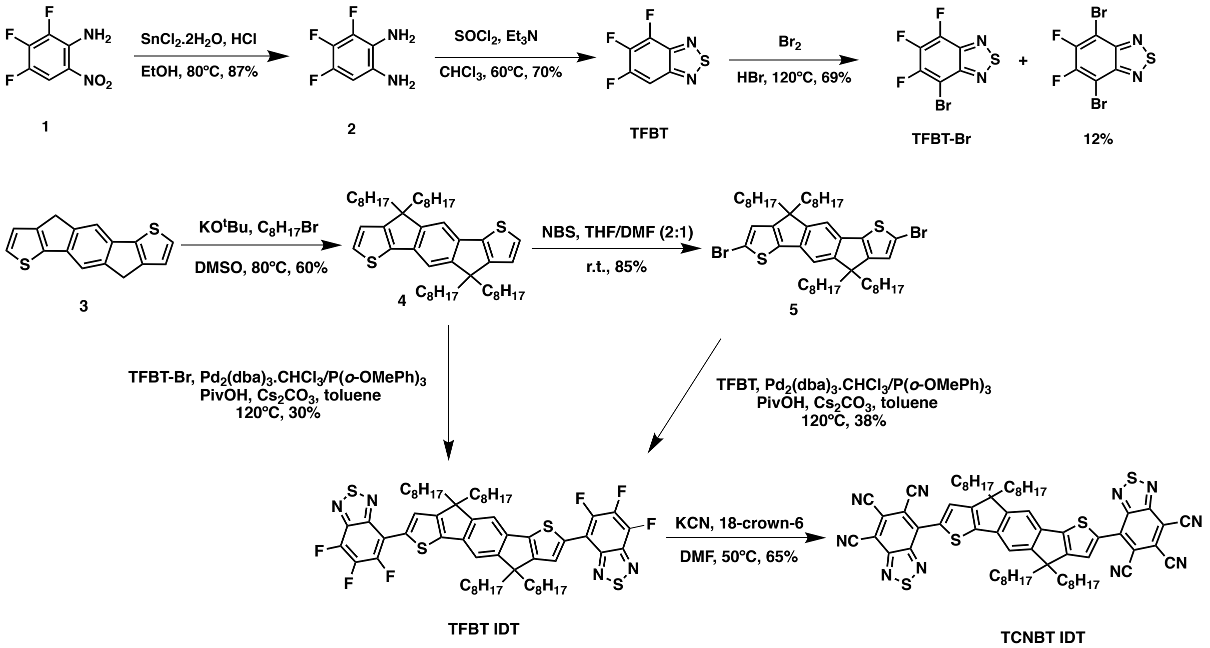

Scheme S1. Synthesis of **TFBT** and **TFBT-Br** (top). Alternative synthesis route to **TFBT IDT** (bottom).

4-Bromo-5,6,7-trifluoro-2,1,3-benzothiadiazole (TFBT-Br): To a suspension of **TFBT** (7.0 g, 36.8 mmol) in HBr (200 mL) was added bromine (17.6 g, 110 mmol) at once. The reaction mixture was heated at 120°C for two to three days. The reaction was monitored by ^{19}F NMR and more bromine was added periodically until consumption of starting material was complete. When no starting material remained, the reaction was left to cool to room temperature. The excess bromine was reduced with sat. Na_2SO_3 and a flaky white solid crashed out, which was filtered and washed with deionised water (~1 L) to afford the brominated product **TFBT-Br** as

an off-white cotton like solid (6.86 g, 25.5 mmol, 69%); m.p. 55-57 °C; ***^{19}F-{H}-NMR** (376 MHz, CDCl$_3$):* δ = -118.61 (dd, J = 19, 5 Hz), -143.18 (dd, J = 19, 5 Hz), -151.54 (dd, J = 19, 5 Hz) ppm; ***HRMS (M^{+}. EI):*** m/z calcd. for $C_6N_2SBrF_3$ 267.8918, found 267.8923.

4,4,9,9-Tetraoctyl-4,9-dihydro-s-indaceno[1,2-b:5,6-b']dithiophene (4): Compound **3** (1.5 g, 5.6 mmol) and potassium tert-butoxide (3.8 g, 33.8 mmol) were purged with nitrogen followed by the addition of degassed, anhydrous DMSO (50 mL). The reaction mixture was stirred at 80°C for one hour. 1-Bromooctane (6.5 g, 33.8 mmol) was then added dropwise and the reaction heated at 80°C for 12 h. The mixture was left to cool to room temperature and the mixture was decanted in 150 mL of water, followed by extraction with hexane (2 x 200 mL) and was further washed with water (100 mL) and brine (100 mL). The organic phase was dried over $MgSO_4$ and the solvent removed under reduced pressure to afford the crude product. Purification using column chromatography (eluent: petroleum ether) yielded a light yellow solid (2.4 g, 3.36 mmol, 60%). m.p. 54 – 56°C; ***^{1}H NMR** (400 MHz, CDCl$_3$):* δ = 7.29 (s, 2H), 7.27 (d, J = 4.9 Hz, 2H), 6.98 (d, J = 4.7 Hz, 1H), 2.02-1.82 (m, 8H), 1.24-1.10 (m, 48H), 0.83 (t, J=7.0 Hz, 12H) ppm; ***^{13}C-{H}-NMR** (CDCl$_3$, 101 MHz):* δ= 155.3, 153.4, 141.8, 135.8, 126.3, 121.9, 113.3, 53.8, 39.4, 32.0, 30.2, 29.5, 29.4, 24.4, 22.8, 14.3 ppm; ***HRMS (M+. EI):*** m/z calcd. for $C_{48}H_{74}S_2$ 714.5231, found 714.5286.

2,7-Dibromo-4,4,9,9-tetraoctyl-4,9-dihydro-s-indaceno[1,2-b:5,6-b']bisthiophene (5): To a solution of 4,4,9,9-tetraoctyl-4,9-dihydro-s-indaceno[1,2-b:5,6-b']dithiophene **4** (370 mg, 0.52 mmol) in THF (8.8 mL) and DMF (4.4 mL) was added N-bromosuccinimide (204 mg, 1.14 mmol) under nitrogen. The reaction was stirred at r.t. for 30 min in the absence of light. After reaction completion, which was monitored by TLC (eluent: petroleum ether), sat. Na_2SO_3 was added followed by addition of water (50 mL). The product was extracted with hexane (2 x 50 mL) and dried over $MgSO_4$. Removal of the solvent under reduced pressure afforded the product as a yellow solid (381 mg, 0.44 mmol, 85 %). m.p. 96 – 98°C; ***^{1}H NMR** (CDCl$_3$, 400 MHz):* δ = 7.17 (s, 2H), 6.96 (s, 2H), 1.96 – 1.77 (m, 8H), 1.22 – 1.09 (m, 48H), 0.82 (t, J = 7.0 Hz, 12H) ppm; ***^{13}C-{H}-NMR** (CDCl$_3$, 101 MHz)* δ = 154.2, 152.3, 142.0, 135.6, 130.0, 128.8, 124.9, 113.1, 112.5, 54.9, 39.1, 31.9, 30.1, 29.9, 29.4, 29.4, 24.3, 22.8, 14.2 ppm; ***HRMS (M+. EI)*** m/z calcd. for $C_{48}H_{72}Br_2S_2$ 870.34, found 870.3425.

Alternative procedure for the synthesis of 4,4'-(4,4,9,9-Tetraoctyl-4,9-dihydro -s-indaceno [1,2-b:5,6-b'] bisthiophene -2,7-diyl) bis (5,6,7-trifluoro-2,1,3-benzothiadiazole) (TFBT IDT). Compound **4** (300 mg, 0.34 mmol), **TFBT-Br** (277 mg, 1.03 mmol), $Pd_2(dba)_3 \cdot CHCl_3$ (18 mg, 5%), tris(*o*-anisyl)phosphine (10 mg, 0.034 mmol), pivalic acid (10 mg, 0.10 mmol) and caesium carbonate (332 mg, 1.02 mmol) were added in a sealed vial and

purged with nitrogen. Degassed, anhydrous toluene (5 mL) was added and the mixture was heated to 120°C for 12 h. After cooling, toluene was removed under reduced pressure and the residue was dissolved in dichloromethane (100 mL). The organic phase was washed with water (100 mL) and brine (100 mL) and the crude product was purified using column chromatography [eluent: hexane/ CH_2Cl_2 3:1 (v:v)] and further purified by recycling GPC in chloroform. The product was isolated as deep red solid (110 mg, 0.10 mmol, 30%); m.p. (DSC) 148-149°C; ***^{1}H NMR*** *(400 MHz, $CDCl_3$):* δ = 8.14 (s, 2H), 7.44 (s, 2H), 2.14-1.92 (m, 8H), 1.12-0.90 (m, 48H), 0.78 (t, J = 7.1 Hz, 12 H) ppm; ***^{19}F-{H}-NMR*** *(376 MHz, CDCl3): δ* = -127.17 (d, J = 16 Hz), -146.49 (d, J = 16 Hz), -153.28 (t, J = 16 Hz) ppm; UV/Vis ($CHCl_3$): λ_{max} (ε): 315 nm (31268 M^{-1} cm^{-1}), 388 nm (58475), 516 nm (66980); ***MS (MALDI-TOF)*****:** isotopic cluster at m/z = 1091 [M+].

3. Optoelectronic characterization of TFBT IDT and TCNBT IDT

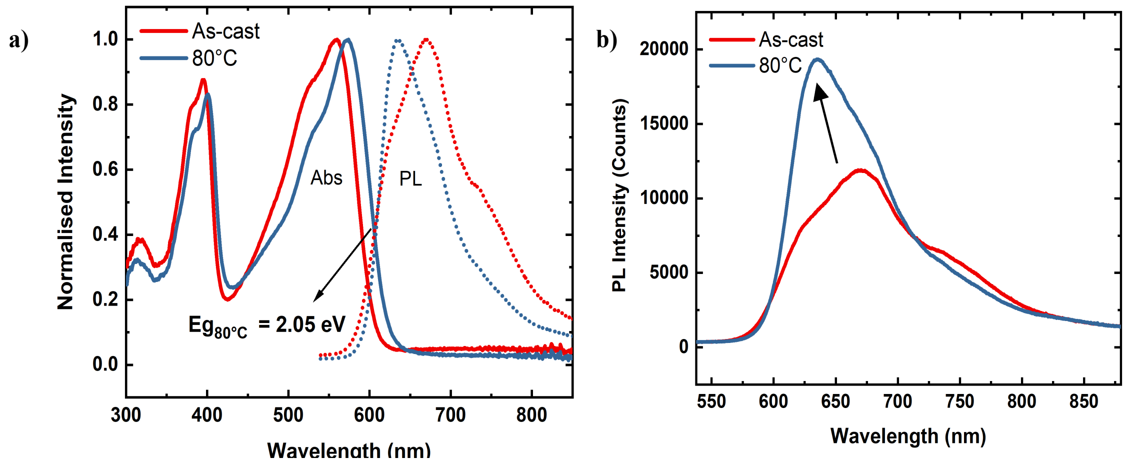

Figure S2. a) Normalized absorption and photoluminescence spectra of **TFBT IDT**. The Stokes shift is 110 nm (0.36 eV) and 58 nm (0.20 eV) for the as-cast and 80°C annealed samples, respectively. The bandgap (Eg) for the as-cast film (2.08 eV) and 80°C annealed film (2.05 eV) was calculated by means of the intersection wavelength of the absorption and photoluminescence spectra. The arrow shows the intersection for the 80°C annealed film. **b)** Photoluminescence spectra of **TFBT IDT** as cast and after annealing at 80°C showing an increase in the absolute photoluminescence upon annealing.

a)

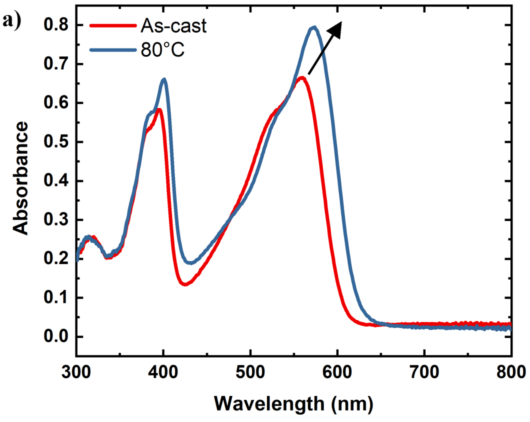

b)

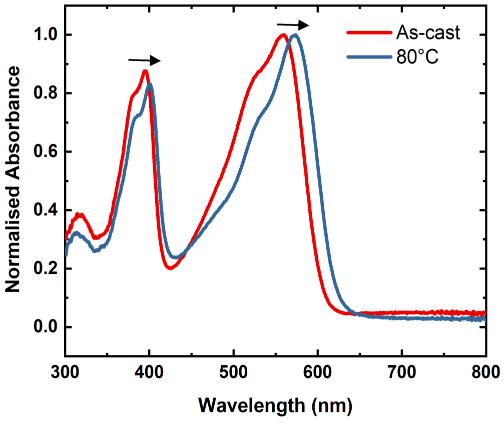

Figure S3. **a)** UV-vis absorption spectra of **TFBT IDT** showing a redshift upon annealing with an increase in the absolute absorption of **TFBT IDT** films, indicated with an arrow. The absorption onset is 2.05 eV and 1.99 eV for as-cast and 80°C annealed samples, respectively. **b)** Normalized UV-vis absorption spectra of **TFBT IDT** for thin films (as-cast and annealed at 80°C). Both peaks redshift by the same energy of approximately 0.055 eV.

a)

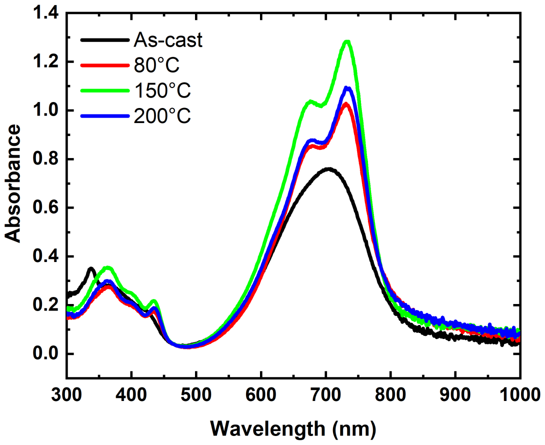

b)

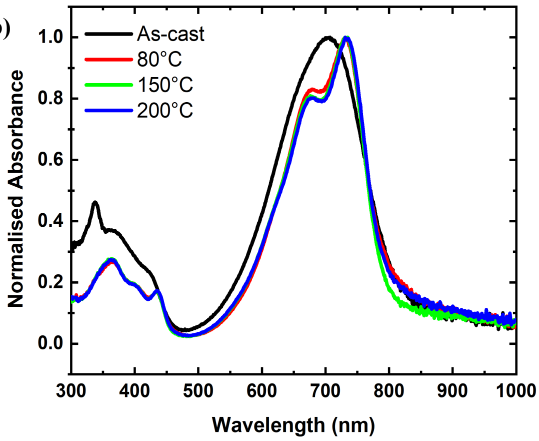

Figure S4. UV-vis absorption spectra of thin films annealed at different temperatures, showing the effect of different annealing temperatures on the absorption of **TCNBT IDT** films. The onset is almost unchanged at 1.55 ± 0.02 eV.

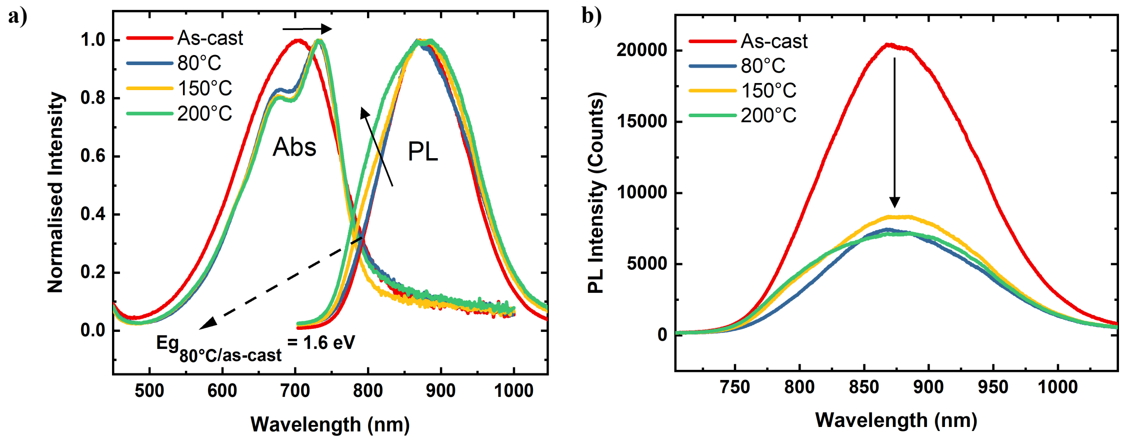

Figure S5. a) Normalized absorption and photoluminescence spectra of **TCNBT IDT**. Bandgap (Eg) calculation in the solid-state for as-cast and 80°C annealed films from the intersection wavelength of the absorption and photoluminescence spectra. The Stokes shift is 167 nm (0.33 eV) and 142 nm (0.27 eV) for the as-cast and annealed samples, respectively. **b)** Photoluminescence spectra of **TCNBT IDT** as cast and after annealing at different temperature showing quenching of the photoluminescence by annealing, consistent with the increase in crystallinity.

4. Determination of the energy levels of TFBT IDT and TCNBT IDT

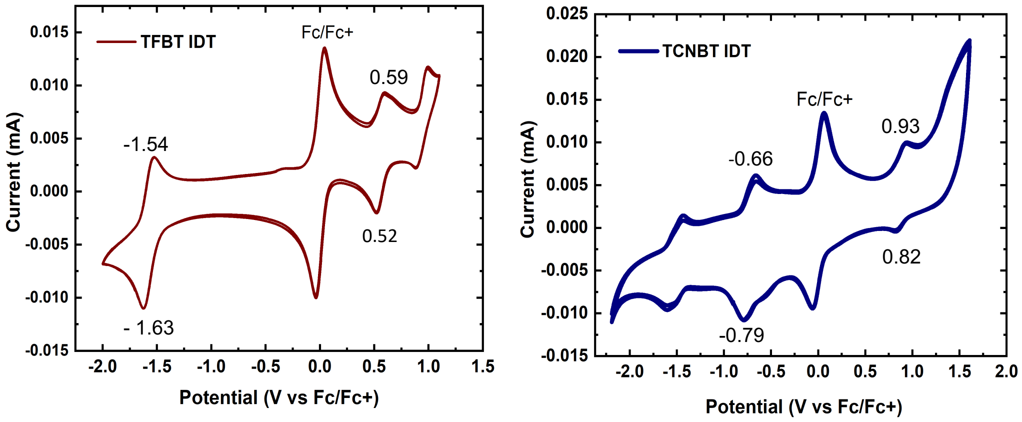

Figure S6: Cyclic voltammograms of **TFBT IDT** (left) and **TCNBT IDT** (right) in a dichloromethane-[n-Bu_4N]PF_6 solution (0.1 M) at a 100 mVs^{-1} scan rate; the potentials were measured against a Ag/AgCl reference electrode. The potentials were referenced by using ferrocene (shown as Fc/Fc^+ on each graph) as an internal standard, and any solvent effects were neglected. E_a and E_c values are indicated for both compounds.

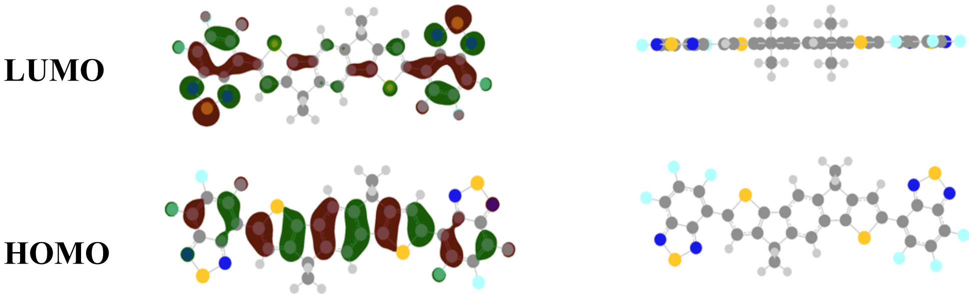

Figure S7. Orbital visualizations of the minimized energy structures of **TFBT IDT**, simulated using DFT at the B3LYP level of theory with the 6-31G(d,p) basis set. **HOMO** (bottom) at -0.18794 Hartrees (-5.1 eV) and **LUMO** (top) at -0.10587 Hartrees (-2.9 eV).

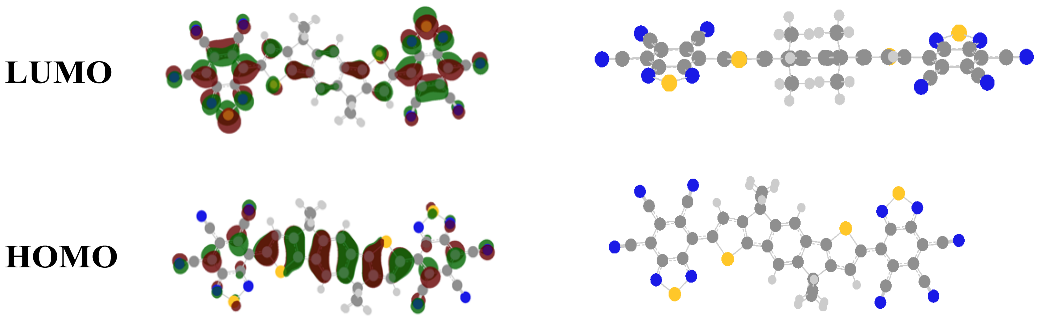

Figure S8. Orbital visualizations of minimized energy structures of **TCNBT IDT**, simulated using DFT at the B3LYP level of theory with the 6-31G(d,p) basis set. **HOMO** (bottom) at -0.22257 Hartrees (-6.1 eV) and LUMO (top) at -0.15182 Hartrees (-4.1 eV). The optimized structure has a dihedral of approximately 20°.

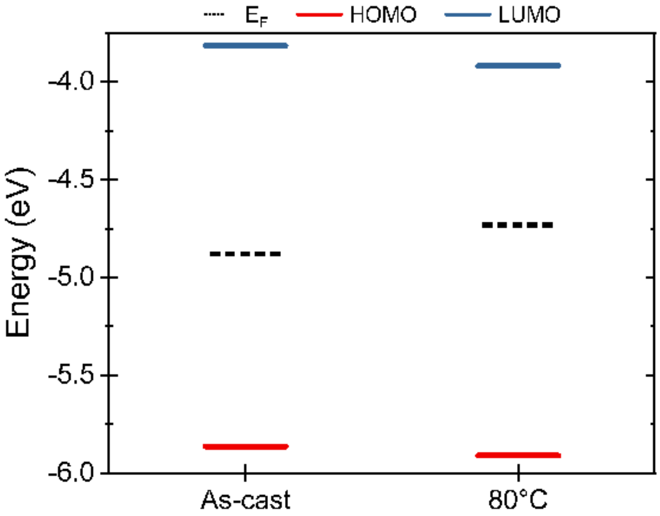

Figure S9. Kelvin probe (KP) and air photoemission spectroscopy (APS) measurements of **TFBT IDT** on ITO, as-cast and annealed at 80°C (measurement error ± 0.05 eV). The Fermi level (E_F) is shallower after annealing. The LUMO energy level was calculated by the addition of the optical band gap (the intersection wavelength of the absorption and photoluminescence spectra (**Figure S2a**)).

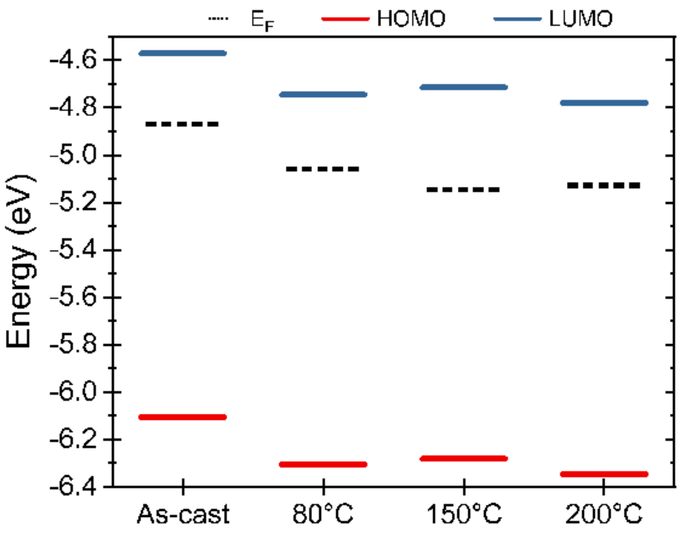

Figure S10. a) KP and APS measurements of **TCNBT IDT** for different films on ITO at annealing temperatures. APS values indicate deepening of HOMO energy levels (measurement error ±0.05 eV) and increased crystallinity. The Fermi level also deepens following the same trend. The LUMO energy level was calculated by the addition of the optical band gap (obtained from the intersection wavelength of the absorption and photoluminescence spectra (**Figure S5 a)**).

5. Thermogravimetric analysis (TGA)

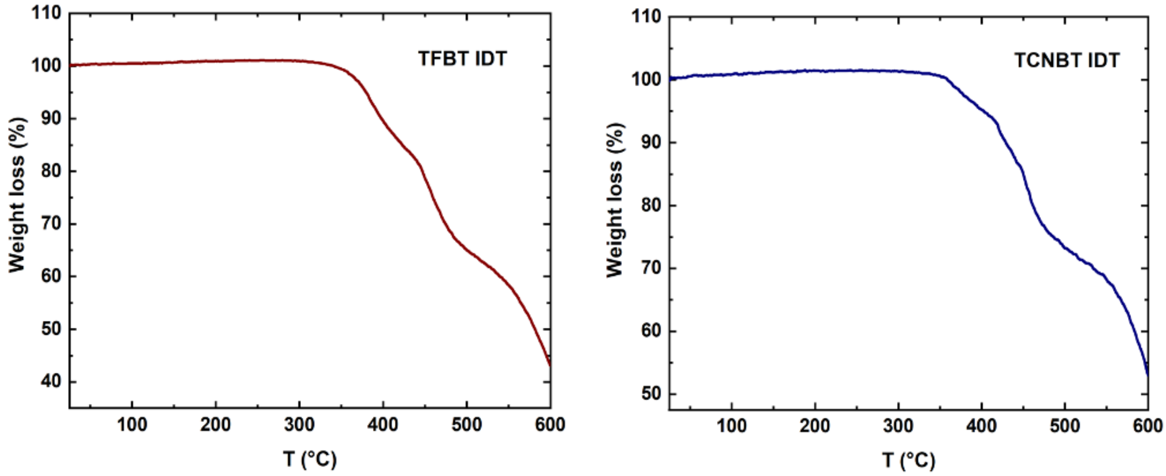

Figure S11. TGA of **TFBT IDT** (left) and **TCNBT IDT** (right). 5% weight loss for **TFBT IDT** at 380°C and for **TCNBT IDT** at 400°C. Temperature range: 25.0-600°C, with heating at 10.0 K/min in air.

6. Differential scanning calorimetry (DSC)

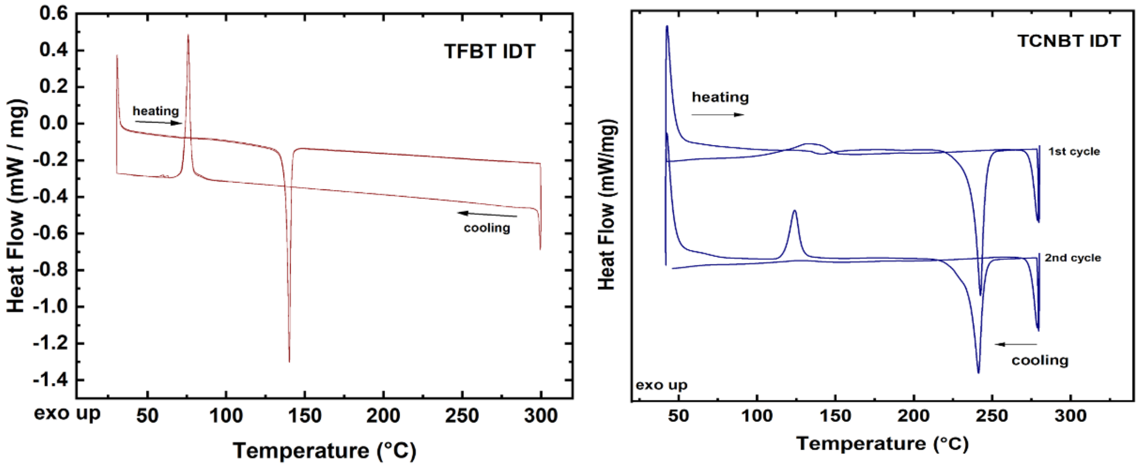

Figure S12. DSC heating and cooling curves for **TFBT IDT** (left) with a scan rate of 10°C/min and **TCNBT IDT** (right) with a scan rate of 20°C/min.

7. Stability test for TCNBT IDT

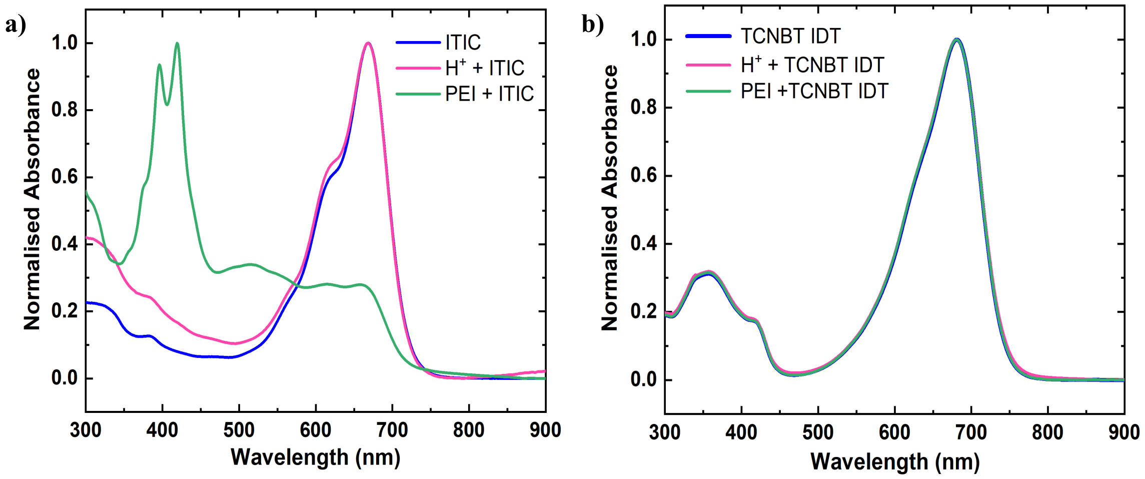

Figure S13. **ITIC** vs **TCNBT IDT** chemical stability in chlorobenzene solution **a)** ITIC (blue) when treated with 0.005 mL acetic acid (pink) and linear 0.1 wt% polyethylenimine (PEI) in isopropanol (green) **b) TCNBT IDT** (blue) chemical stability in chlorobenzene solution when treated with 0.005 mL acetic acid/HCl (pink) and linear 0.1 wt% polyethylenimine (PEI) in isopropanol (green).

8. Structural characterization of TCNBT IDT

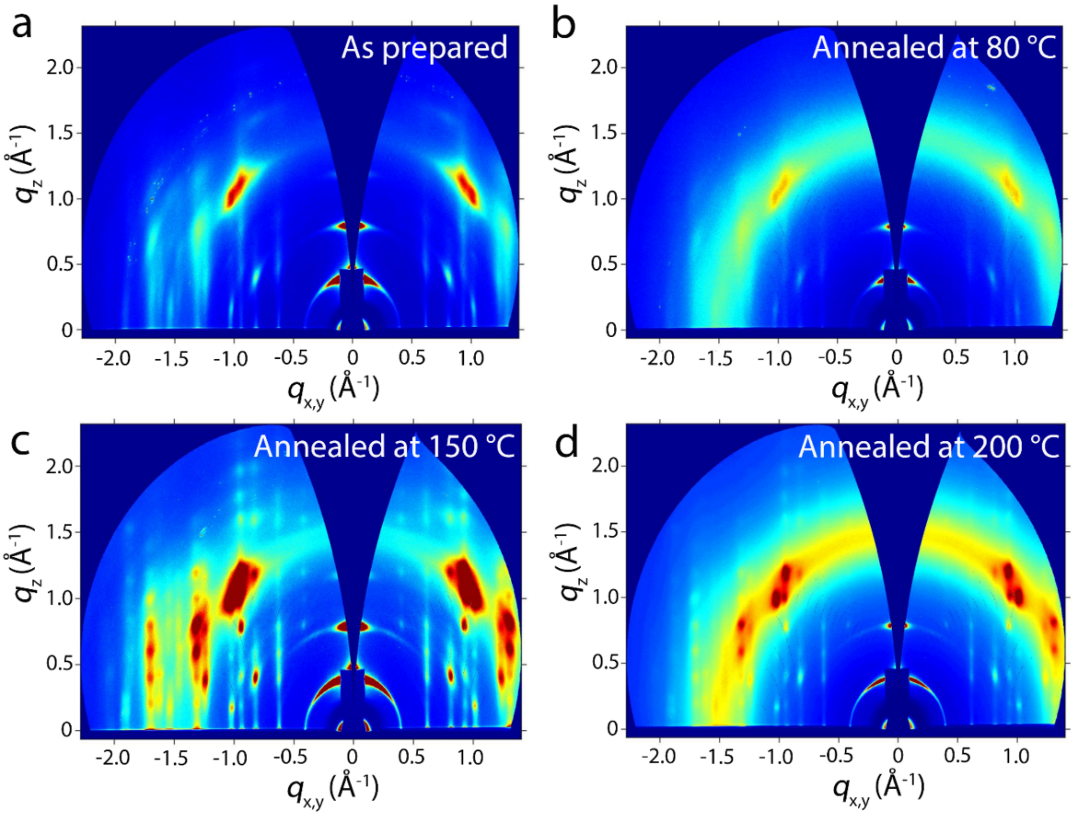

Figure S14. 2-D GIWAXS patterns of **TCNBT IDT** films; a) as prepared, b) annealed at 80°C, c) annealed at 150°C and d) annealed at 200°C.

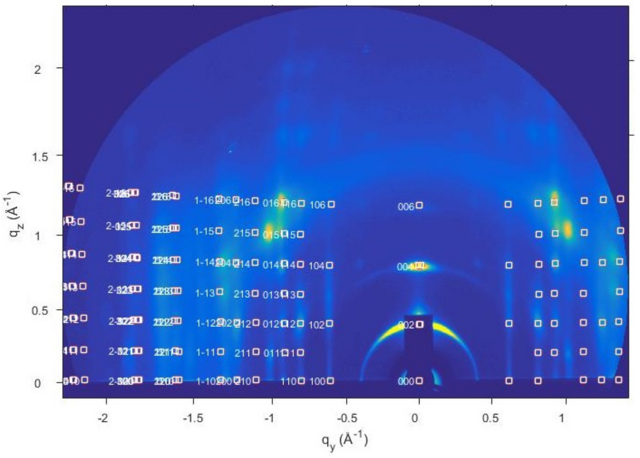

q_{xy} (Å^{-1})	q_z (Å^{-1})	*d* spacing (Å)	Peak Indexing
0.6216	0.0172	10.101	(100)
0.9280	0.0242	6.7684	(010)
0.8145	0.0154	7.7128	(110)
0	0.3949	15.910	(002)

Parameters	NK099
a(Å)	11.7165
b(Å)	7.8505
c(Å)	31.8297
α(deg.)	90
β(deg.)	90
γ(deg.)	60
Space group	$P2_1/c$

Crystal system : Monoclinic
Assemble structure : Brick wall

Figure S15. Molecular diffraction simulation based on the 2-D GIWAXS patterns of **TCNBT IDT** film annealed at 150°C and determination of crystalline structure.

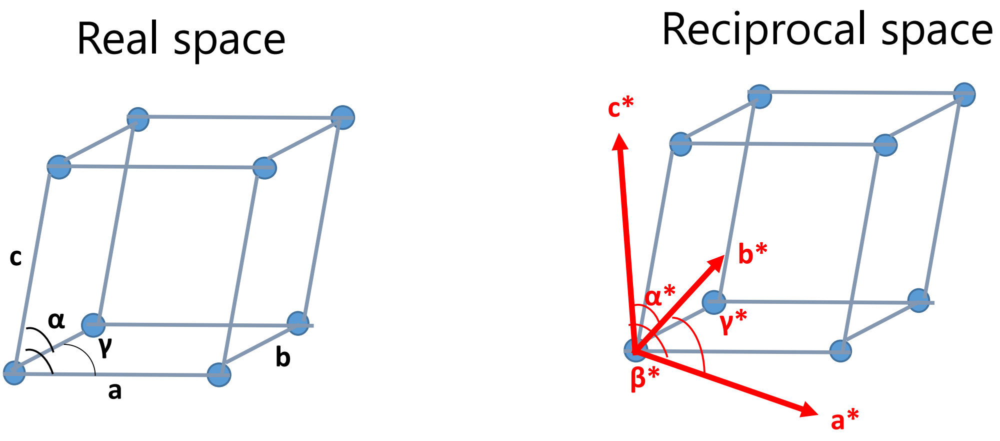

$$a^* = 2\pi \frac{b \times c}{a \cdot (b \times c)} \qquad b^* = 2\pi \frac{c \times a}{a \cdot (b \times c)} \qquad c^* = 2\pi \frac{a \times b}{a \cdot (b \times c)}$$

$$a^* = 2\pi \frac{b \times c}{a \cdot (b \times c)} \qquad cos(\alpha^*) = \frac{cos(\beta)cos(\gamma) - cos(\alpha)}{\sin(\beta) \cdot sin(\gamma)}$$

$$b^* = 2\pi \frac{c \times a}{a \cdot (b \times c)} \qquad cos(\beta^*) = \frac{cos(\gamma)cos(\alpha) - cos(\beta)}{\sin(\gamma) \cdot sin(\alpha)}$$

$$c^* = 2\pi \frac{a \times b}{a \cdot (b \times c)} \qquad cos(\gamma^*) = \frac{cos(\alpha)cos(\beta) - cos(\gamma)}{\sin(\alpha) \cdot sin(\beta)}$$

$$q_{hkl} = ha^* + kb^* + lc^*$$

Figure S16: Lattice parameters: real space vs reciprocal space and definition of lattice parameters.

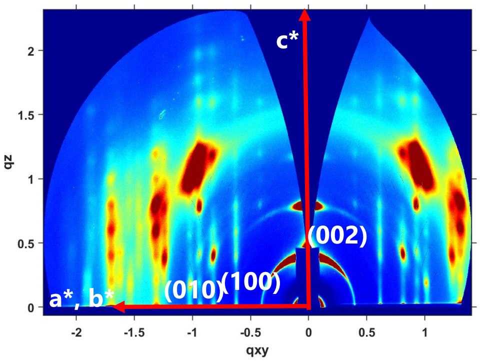

Monoclinic crystal system

$$q_{100} = a* = \frac{2\pi}{a\sin(\gamma)}$$

$$q_{010} = b* = \frac{2\pi}{b\sin(\gamma)}$$

$$q_{002} = 2c* = 2\times\frac{2\pi}{c}$$

$$(q_{110})^2 = (a*)^2 + (b*)^2 + (c*)^2 + 2a^*b^*\cos(\gamma^*)$$
$$= (q_{100})^2 + (q_{010})^2 + (q_{001})^2 + 2q_{100}q_{010}\cos(\gamma^*)$$

$$\cos(\gamma^*) = \frac{1}{2}\cdot\frac{(q_{110}{}^2 - q_{010}{}^2 - q_{100}{}^2)}{q_{010}\cdot q_{100}} = -\cos(\gamma)$$

$q_{hkl} = q_{xy} + q_z$,

where
$q_{xy} = ha^* + kb^*,\ q_z = lc^*$

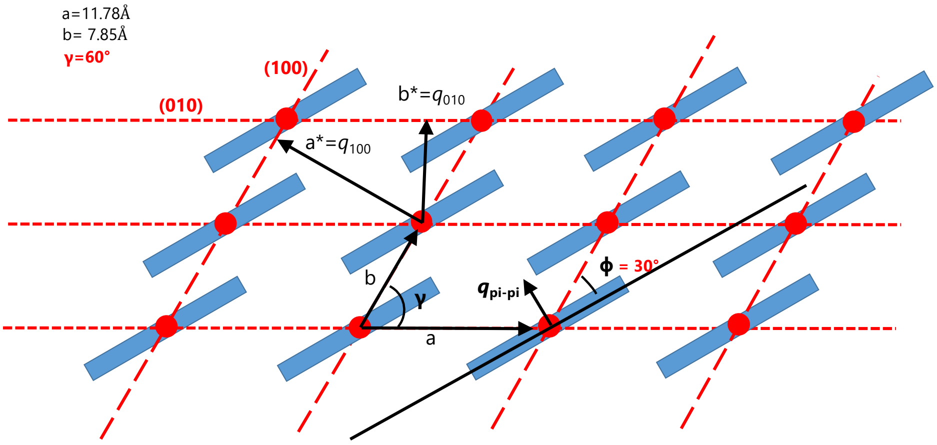

$d_{pi\text{-}pi}$ = b · sin(ϕ),
(ex.: b · sin30° = b/2=3.925 Å)

$q_{pi\text{-}pi}$ =ha*+kb*

$q_{pi\text{-}pi}$ cos(ϕ) = ha*
$q_{pi\text{-}pi}$ cos(γ-ϕ) = kb*

$d_{pi\text{-}pi}$ = b·sin(ϕ)
$q_{pi\text{-}pi}$ =2π/$d_{pi\text{-}pi}$
a*= 2π/a·sin(γ)
b*= 2π/b·sin(γ)

$q_{pi\text{-}pi}$ cos(ϕ) = ka*,
$q_{pi\text{-}pi}$ =2π/$d_{pi\text{-}pi}$ =2π/b·sin(ϕ)
a*= 2π/a·sin(γ)
(2π/b·sin(ϕ)) x cos(ϕ) =h 2π/a·sin(γ)

h= cos(ϕ)*a*sin(γ)/b·sin(ϕ)

$q_{pi\text{-}pi}$ cos(γ-ϕ) = kb*,
$q_{pi\text{-}pi}$ =2π/$d_{pi\text{-}pi}$ =2π/ b·sin(ϕ)
b*= 2π/b·sin(γ)
(2π/ b·sin(ϕ)) x cos(γ-ϕ) =k 2π/b·sin(γ)

k= cos(γ-ϕ)*sin(γ)/sin(ϕ)

Figure S17: Molecular diffraction modelling and simulation based on the 2-D GIWAXS patterns of **TCNBT IDT** film annealed at 150°C.

9. Raman-structure changes upon film annealing

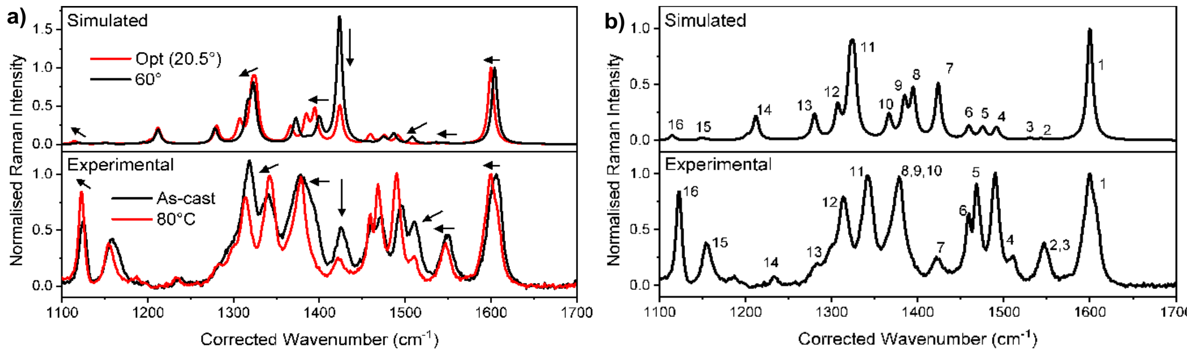

Figure S18. a) (Top) Normalised simulated Raman spectra of **TCNBT IDT** at its minimum energy structure and with an induced dihedral torsion of 60°, (bottom) experimental normalised and baselined Raman spectra taken at 514 nm excitation of **TCNBT IDT** films at different annealing temperatures. Arrows indicate matching peak changes that occur upon moving towards a more planar structure in the simulated spectra and upon annealing in the experimental spectra. The similarity between the changes suggests that a more planar structure is adopted by the molecules upon annealing. **b**) Normalised and baselined Raman spectra of **TCNBT IDT** with peaks numbered for assignment (see **Table S17** below) for simulated spectra (top) and experimental (bottom) spectra taken on an 80°C annealed film at 514 nm excitation.

Table S1: Full Assignment of Raman peaks of **TCNBT IDT** for simulated and experimental spectra taken on an 80°C annealed film at 514 nm excitation.

Peak	Simulated Wavenumber[1] / cm^{-1}	Experimental Wavenumber / cm^{-1}	Unit	Assignment[3]
1	1600	1600	IDT	Core phenyl - unfused
2	1543	1547	IDT	Core phenyl – fused
3	1530	1547	BT	BT phenyl – off axis
4	1492	1490	BT	BT phenyl and interunit C-C
5	1476	1468	Side Chains	Side chain H-wagging
6	1459	1459	IDT & side chains	Predominantly C-C thiophene, some contributions from side chains
7	1423	1423	IDT and BT	Predominantly C-C thiophene, some contribution from BT
8	1394	1378	Side chains	Side chain H-wagging
9	1385	1378	Side chains	Side chain H-wagging
10	1366	1378	BT	BT phenyl – off axis
11	1324[2]	1342	IDT & BT	Core phenyl breathing and cyclopentadiene and CN in thiadiazole
12	1307	1313	IDT	Predominantly C=C thiophene, but also delocalised
13	1280	1298/1283	BT	C-C bond in BT phenyl
14	1233	1212	Side chains and IDT	H-wagging
15	1154	1150	IDT	H-wagging
16	1123	1114	BT	BT C-CN bond

[1] Presented frequencies have had an empirical 0.97 scaling factor applied
[2] Composed of two equally intense modes at 1321 and 1326 cm^{-1}
[3] Assignment is based on visualisation of the vibrational modes in GaussView 6.0.16. Often modes are delocalised with many bonds and atoms contributing to the collective mode, in such instances effort has been made to assign only the most prominent vibration, although some collective contributions are too large to ignore.

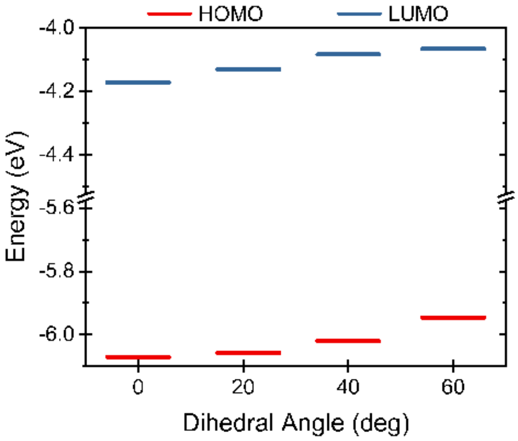

Figure S19: Simulated energy levels as a function of dihedral angle for **TCNBT IDT** showing decrease in HOMO energy with increased planarity.

10. Transistor performance and optimization

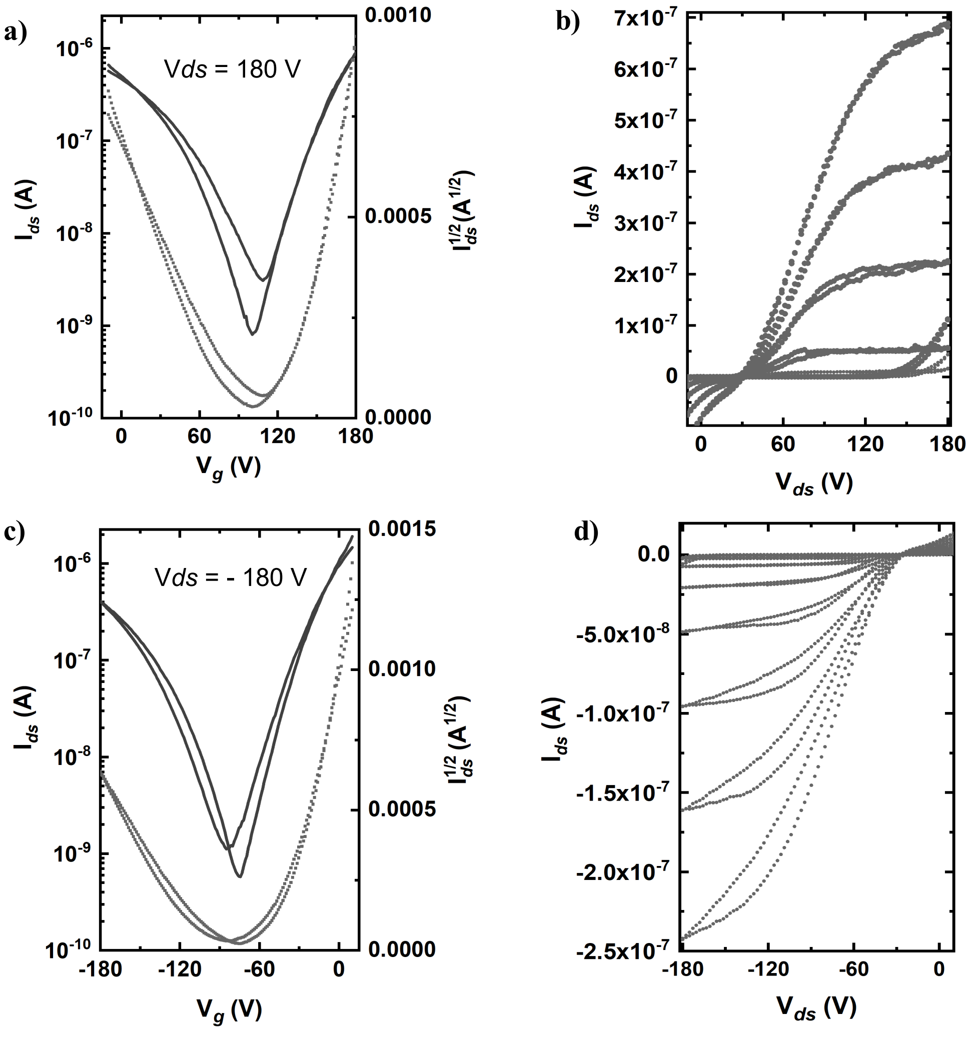

Figure S20. TFBT IDT OFET performance in a BC/TG architecture. **a)** & **b)** n-Type transfer and output curves. **c)** & **d)** p-Type transfer and output curves. For the output measurements gate voltage (Vg) applied from 60 to 180 V or -60 to -180 V (20 steps). The films were annealed at 120°C, and the device dimensions were 1000/30 (W/L).

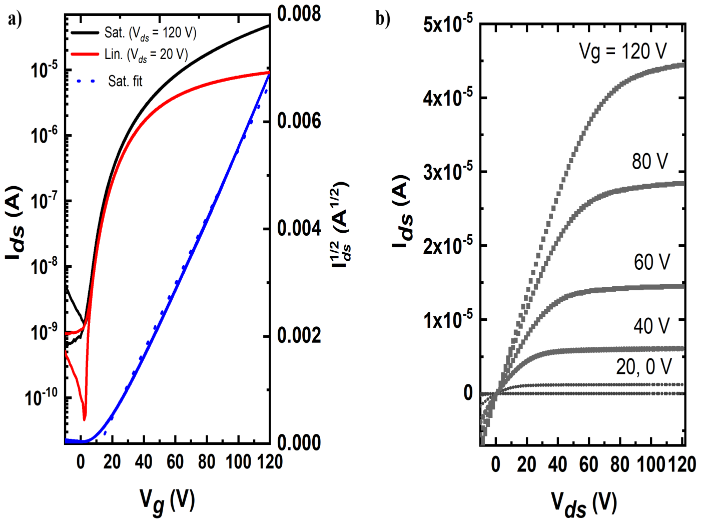

Figure S21. TCNBT IDT OFET performance in a BC/TG architecture: **a)** transfer and **b)** output curves, W/L = 1000/30 µm, μ_{lin} = 0.04 cm^2 V^{-1} s^{-1} μ_{sat} = 0.11 cm^2 V^{-1} s^{-1}, V_T = 5 V and $I_{on/off}$ = 10^4.

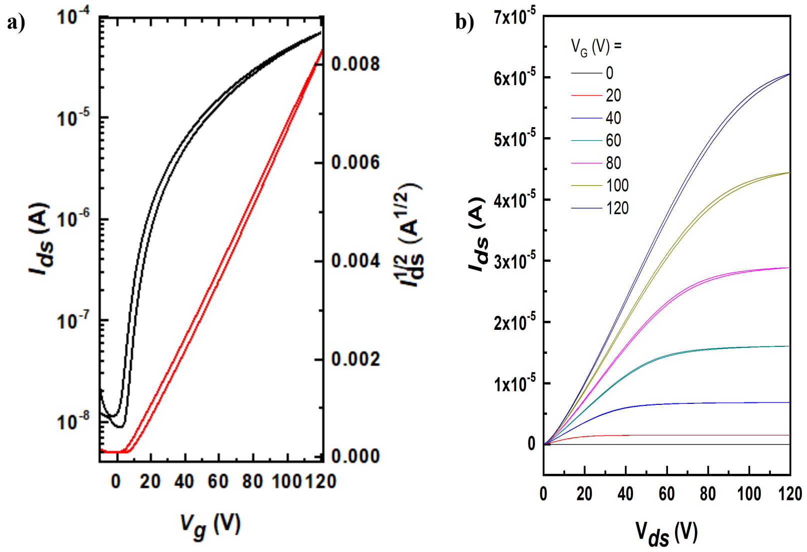

Figure S22. TCNBT IDT OFET performance in a TC/BG architecture with Au (S/D): **a)** transfer and **b)** output curves, W/L = 1000/40 µm, μ_{sat} = 0.051 cm^2 V^{-1} s^{-1}, V_T = 8 V and $I_{on/off}$ = 10^4.

TC/BG Device Optimization - Active layer annealing temperature effect

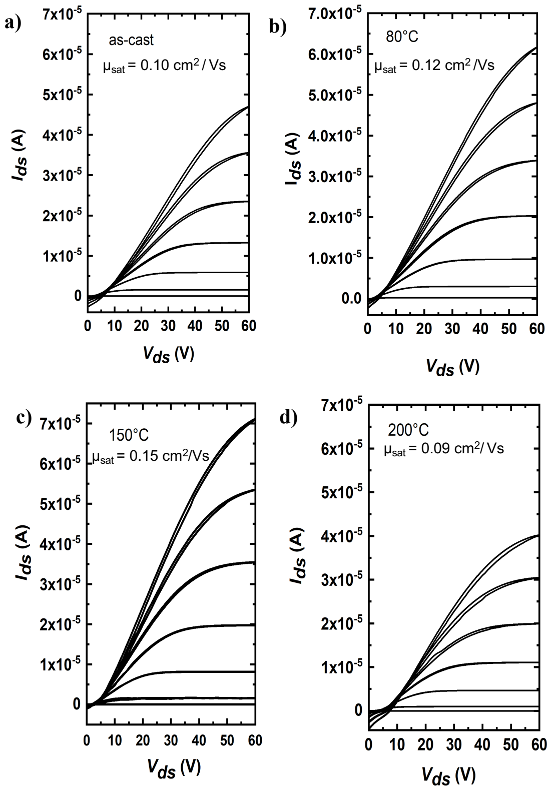

Figure S23. Active layer film annealing temperature dependence of device performance for **TCNBT IDT**. Films **a)** as-cast **b)** 80°C **c)** 150°C and **d)** 200°C annealing temperature output curves. The film annealed at 150°C was the best performing film with the highest drain current, minimum contact resistance and highest saturation mobility value. For the output measurements gate voltage (V_g) was applied from 0 to 60 V (10 steps).

TC/BG Device Optimization – Device annealing effect

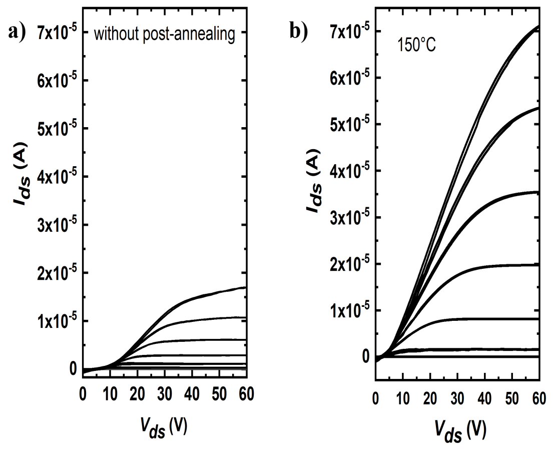

Figure S24. In both figures, the **TCNBT IDT** film was annealed at 150°C. **a)** OFET performance without device annealing and **b)** OFET performance after the device was annealed at 100°C for 3 minutes.

Table S2: Average mobility on optimised OFETs. The films were annealed at 150°C; TC/BG architecture W/L(Al) = 1500/50 µm, BC/TG architecture W/L(Au) = 1000/30 µm, TC/BG W/L(Au) = 1000/40 µm.

TCNBT IDT	$\mu_{sat.\ ave}$ ($\mu_{sat,\ max}$ cm^2 V^{-1} s^{-1})	$I_{on/off}$	V_T (V)
TC/BG Al(S/D) (11 devices)	0.12 ± 0.03 (0.150)	10^5	0.1-0.5
BC/TG Au(S/D) (10 devices)	0.09 ± 0.02 (0.110)	10^3~10^4	3-7
TC/BG Au(S/D) (6 devices)	0.057± 0.008 (0.065)	10^4	8-10

11. TCNBT IDT stability tests

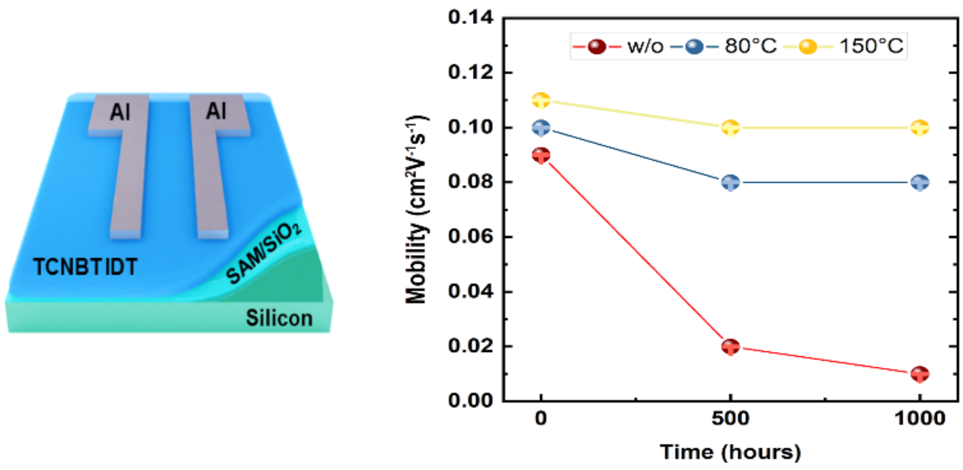

Figure S25. The air stability of **TCNBT IDT** in the solid-state was tested using a top contact/bottom gate architecture. A silicon substrate was treated with octadecyltrichlorosilane (100 µL in 10 mL, 10 minutes at 100°C) and **TCNBT IDT** was spin coated from a chloroform solution (20 mg/mL, 2000 rpm) on top. The films were annealed at different temperatures and left under ambient conditions for 10 weeks [as cast films (w/o-red), 80°C annealed film (blue), 150°C annealed film (yellow). Every 3 weeks, fresh Al contacts were evaporated on top, and the device was measured. As the graph above indicates, **TCNBT IDT** film mobility remained stable for the annealed films but decreased for the as-cast film.

12. NMR spectra

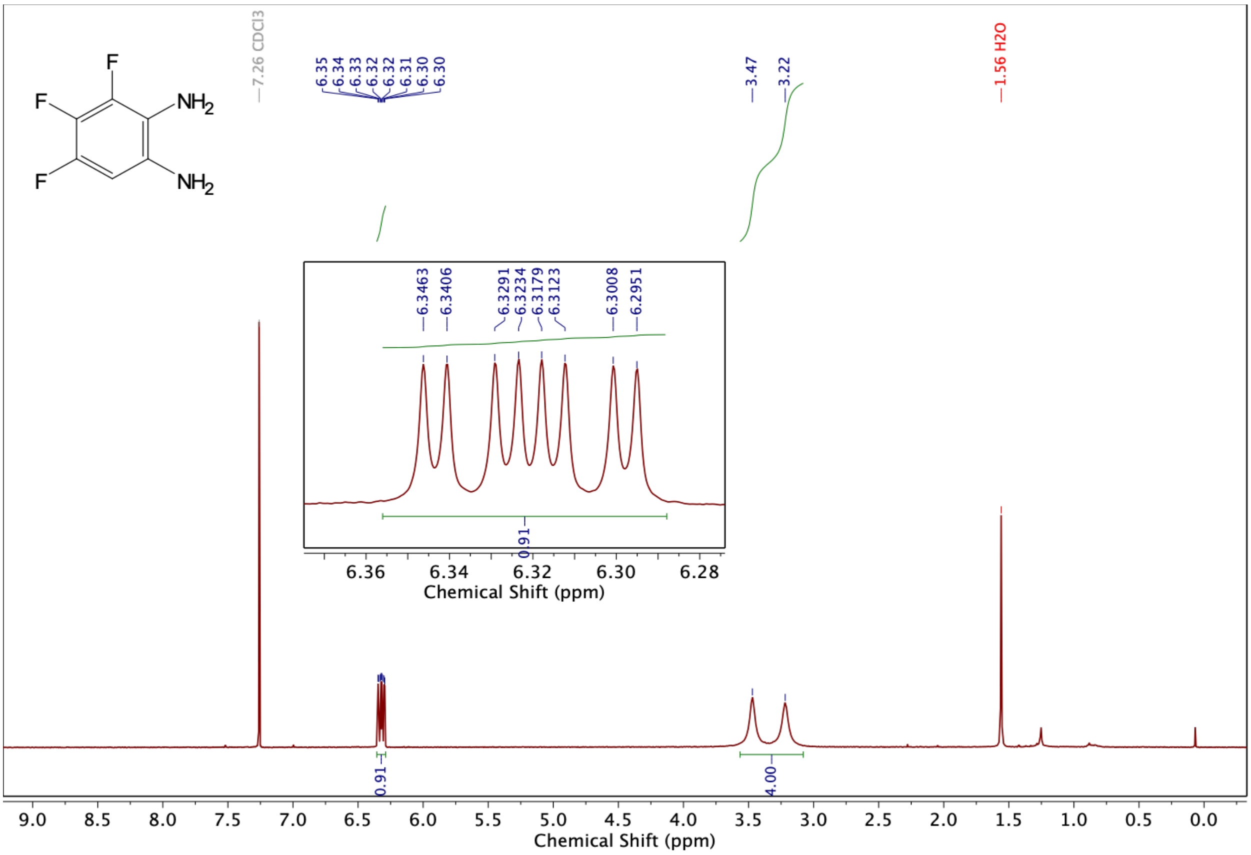

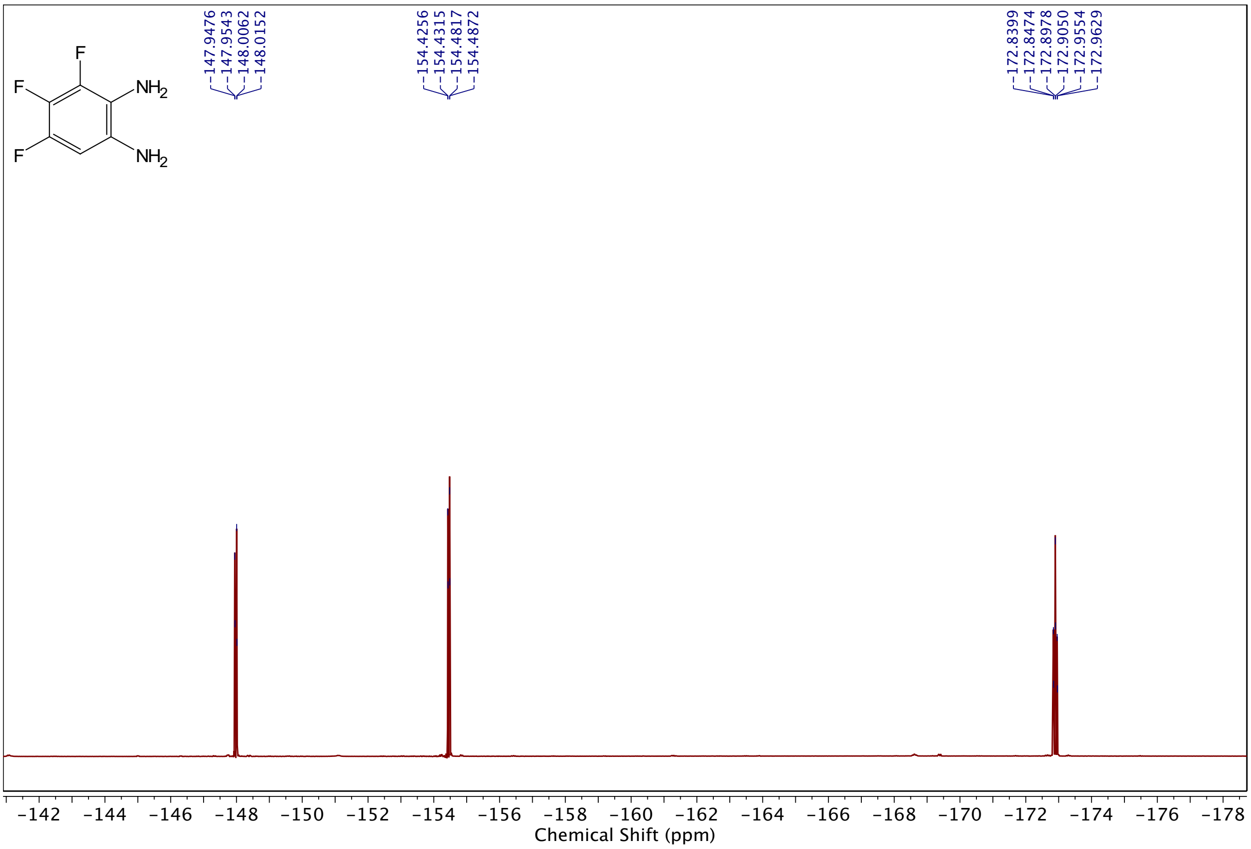

Figure S26. ^{1}H NMR (top) and ^{19}F NMR (bottom) spectra of compound **2** in $CDCl_3$.

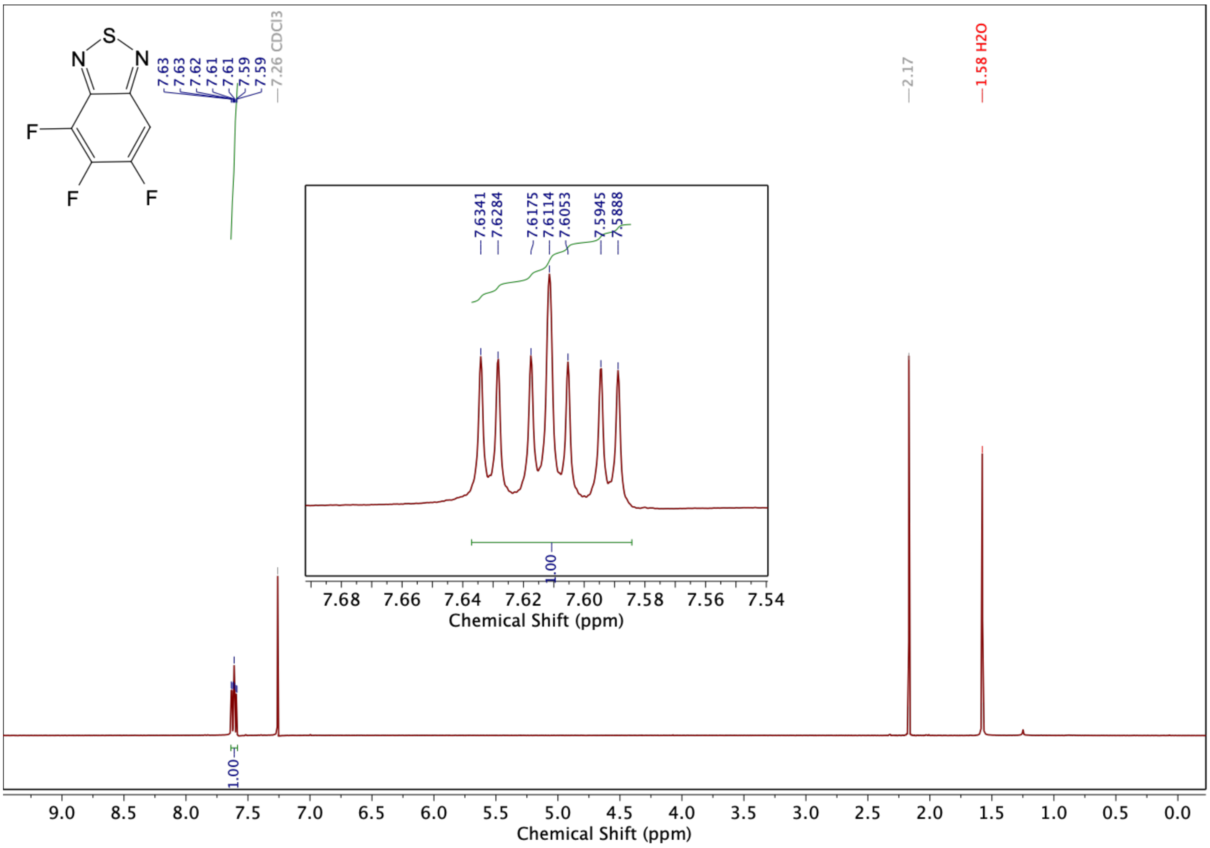

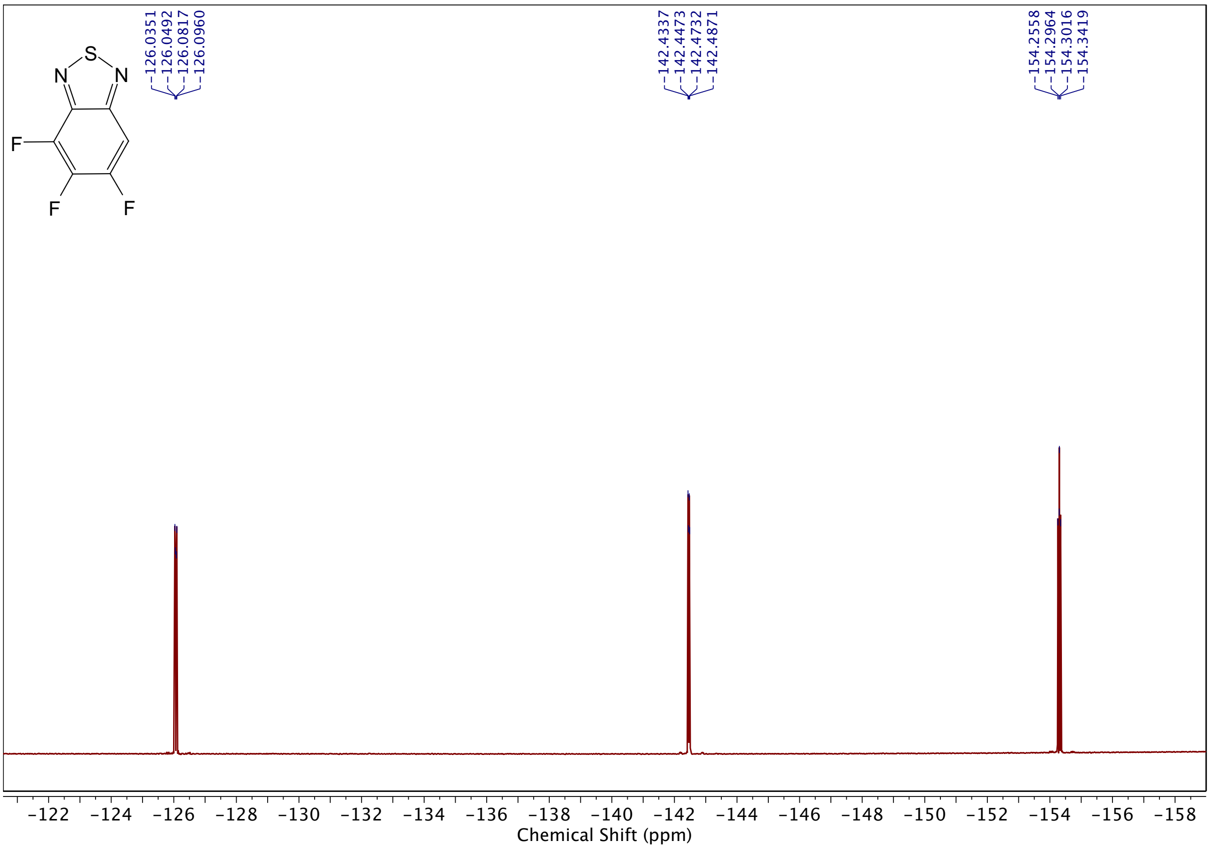

Figure S27. ^{1}H NMR (top) and ^{19}F NMR (bottom) spectra of **TFBT** in $CDCl_3$.

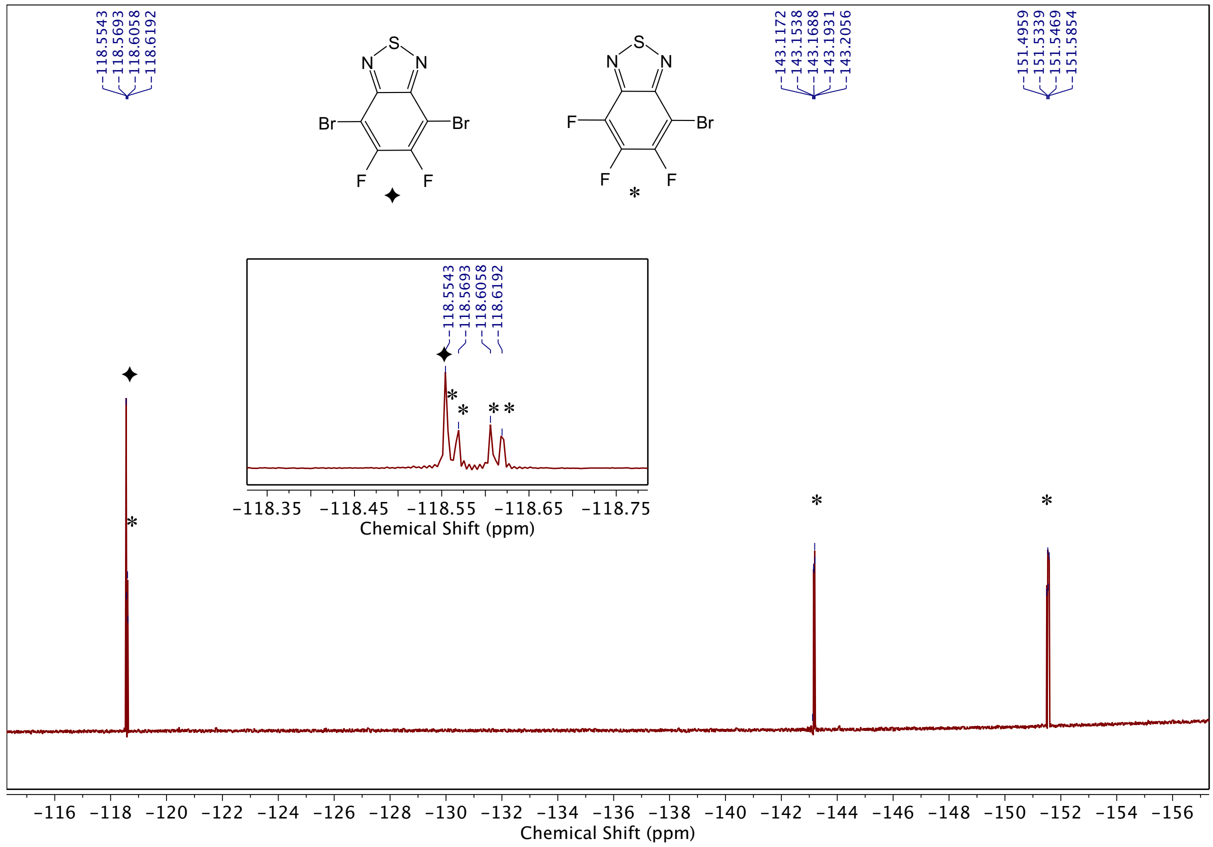

Figure S28. ^{19}F NMR spectrum of **TFBT-Br** (*), highlighting the formation of the dibrominated compound (✦), in $CDCl_3$.

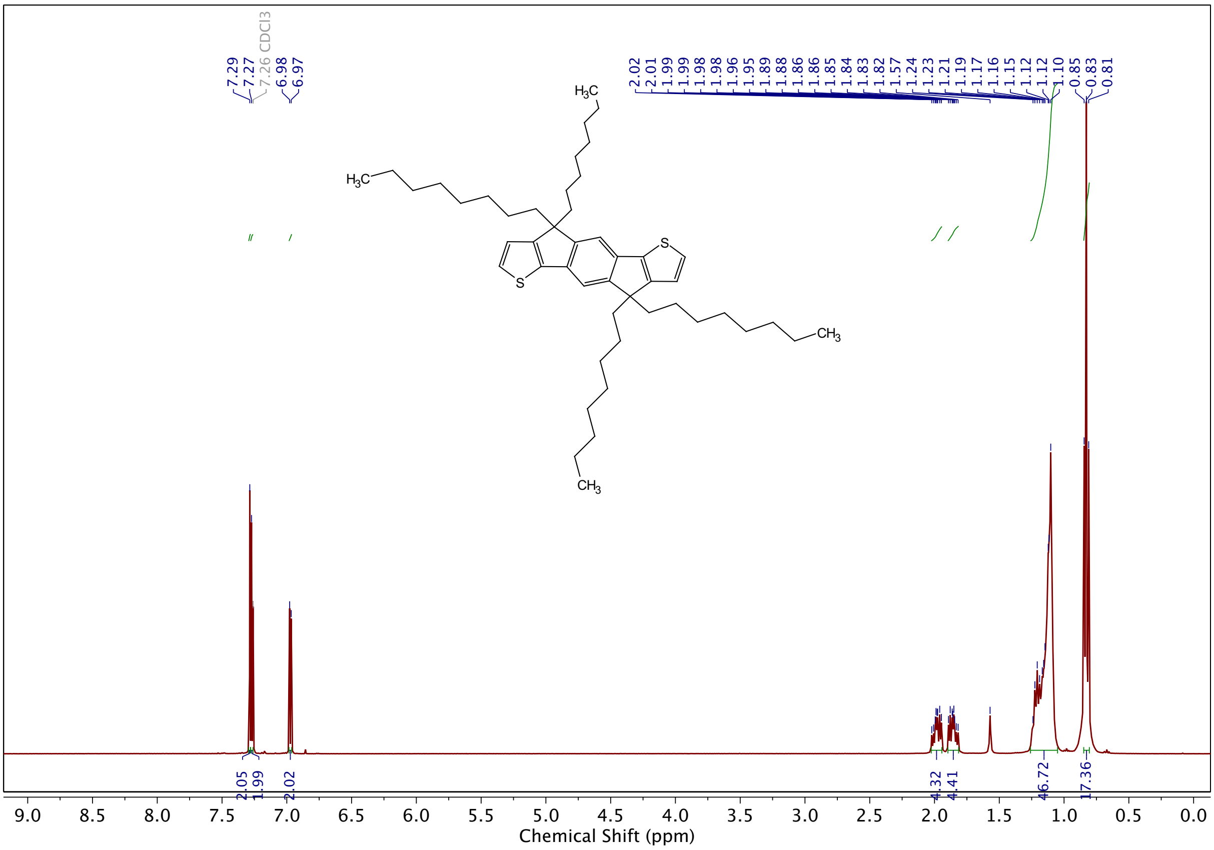

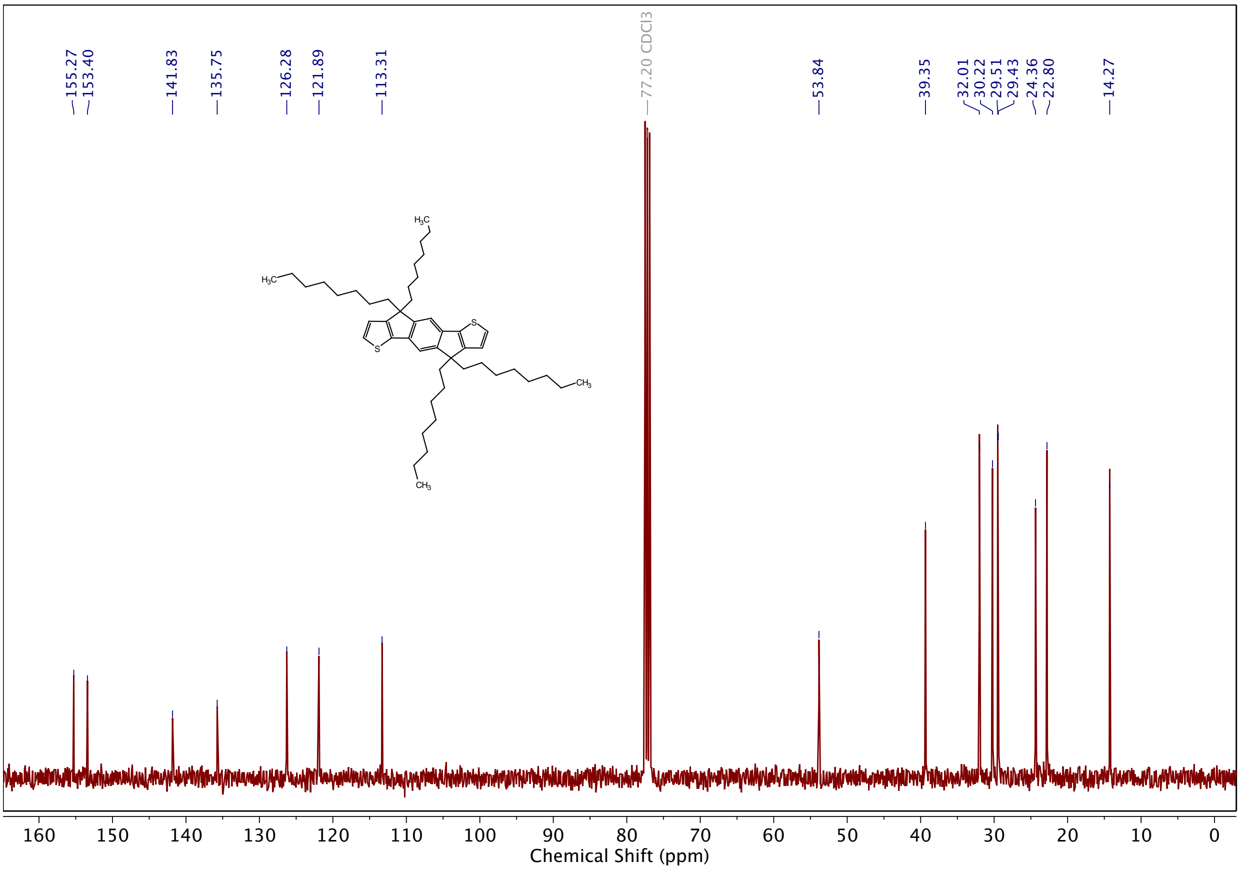

Figure S29. ^{1}H NMR (top) and ^{13}C NMR (bottom) spectra of **4** in $CDCl_3$.

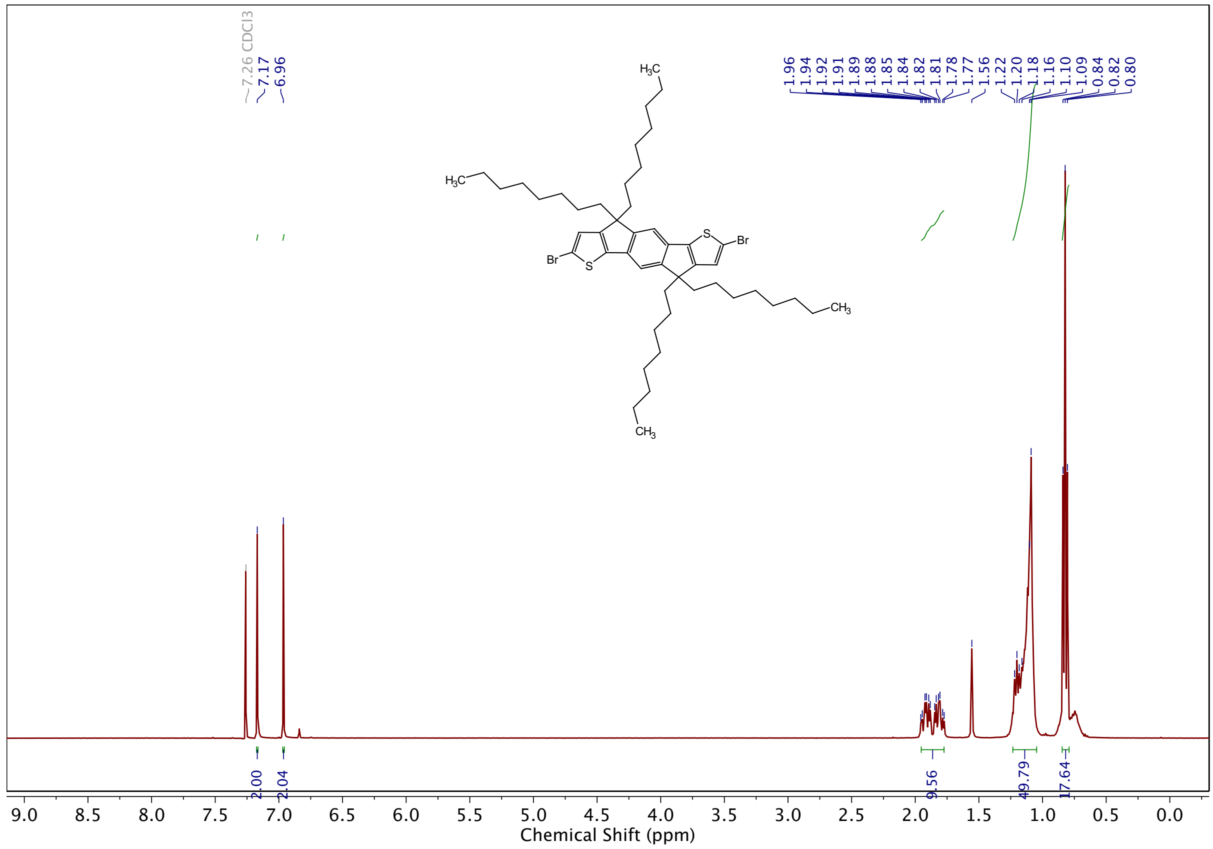

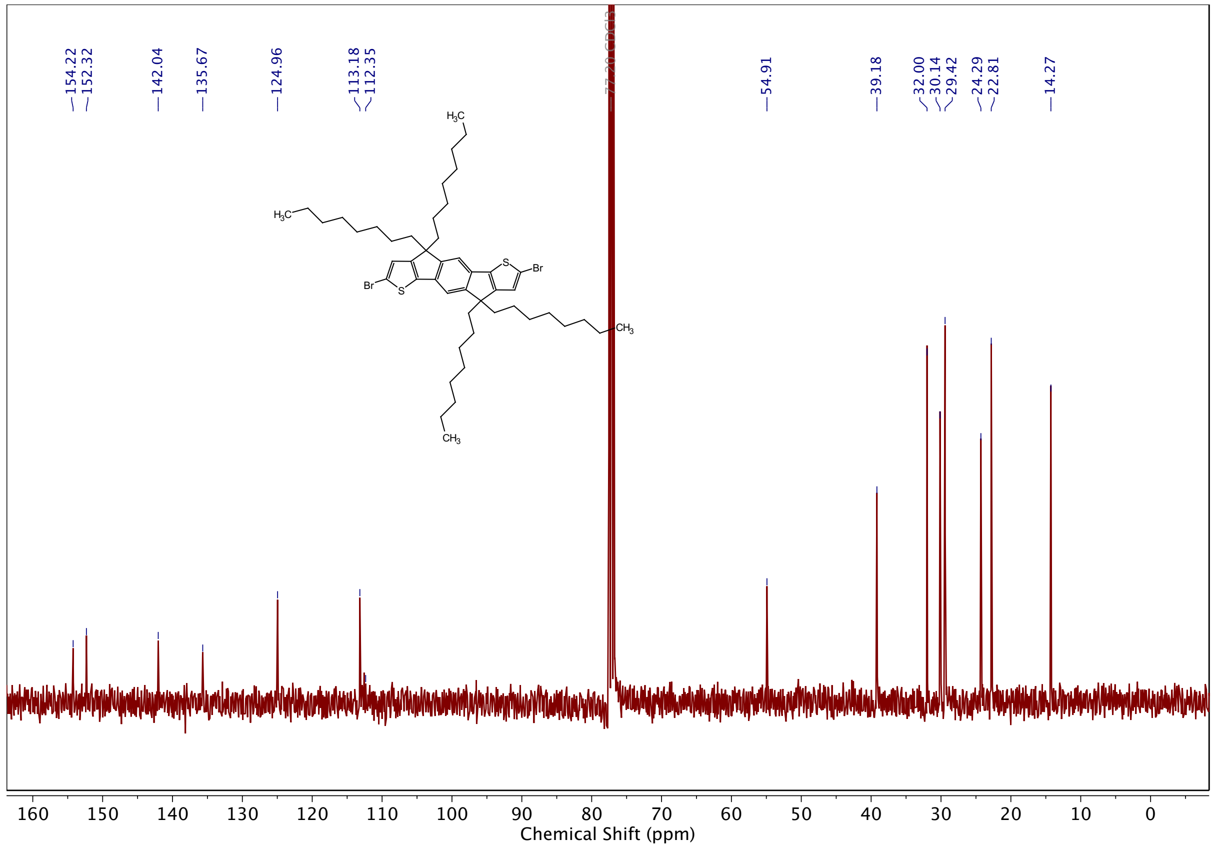

Figure S30. ^{1}H NMR (top) and ^{13}C NMR (bottom) spectra of **5** in $CDCl_3$.

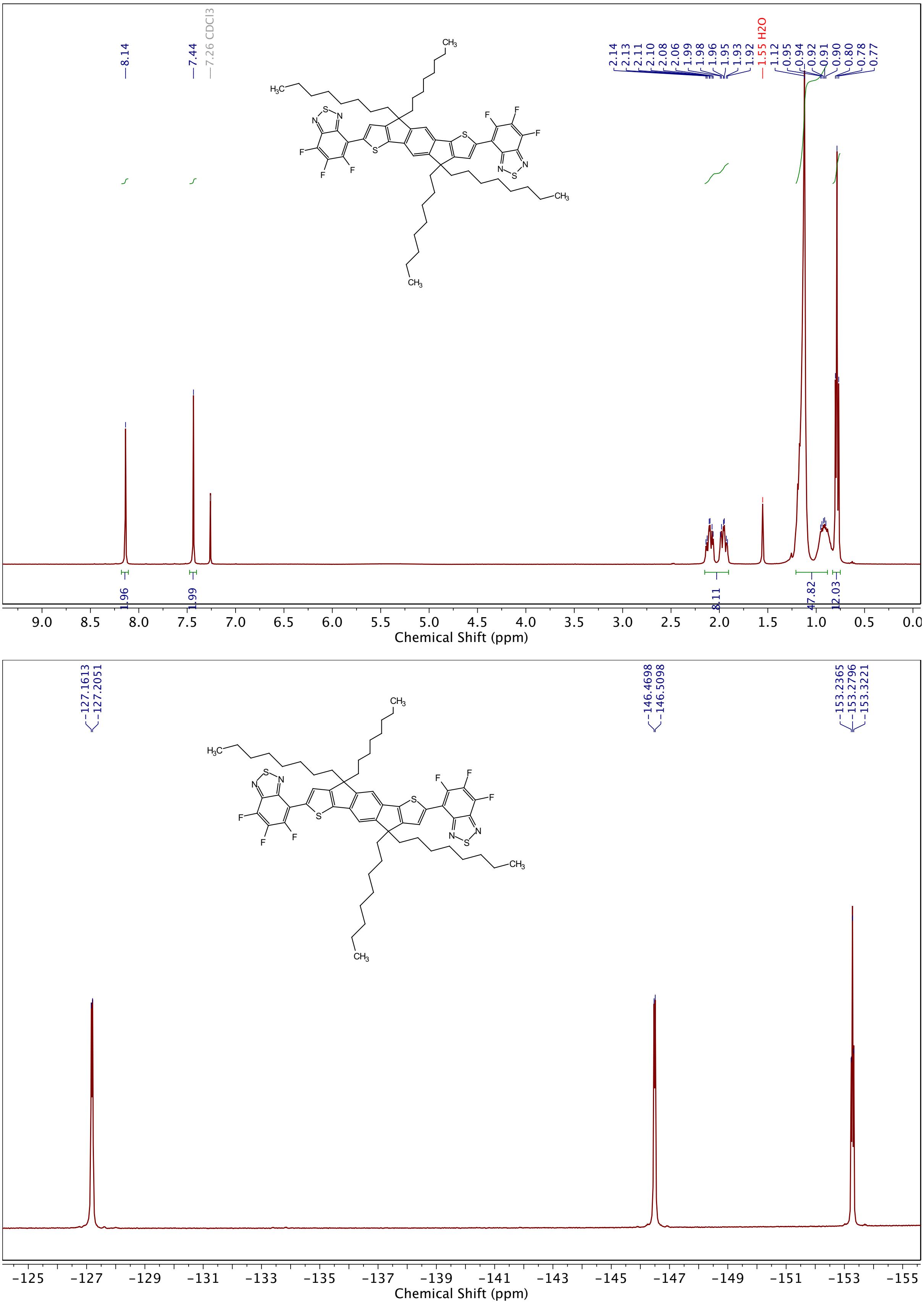

Figure S31. ^1H NMR (top) and ^{19}F NMR (bottom) spectra of **TFBT IDT** in $CDCl_3$.

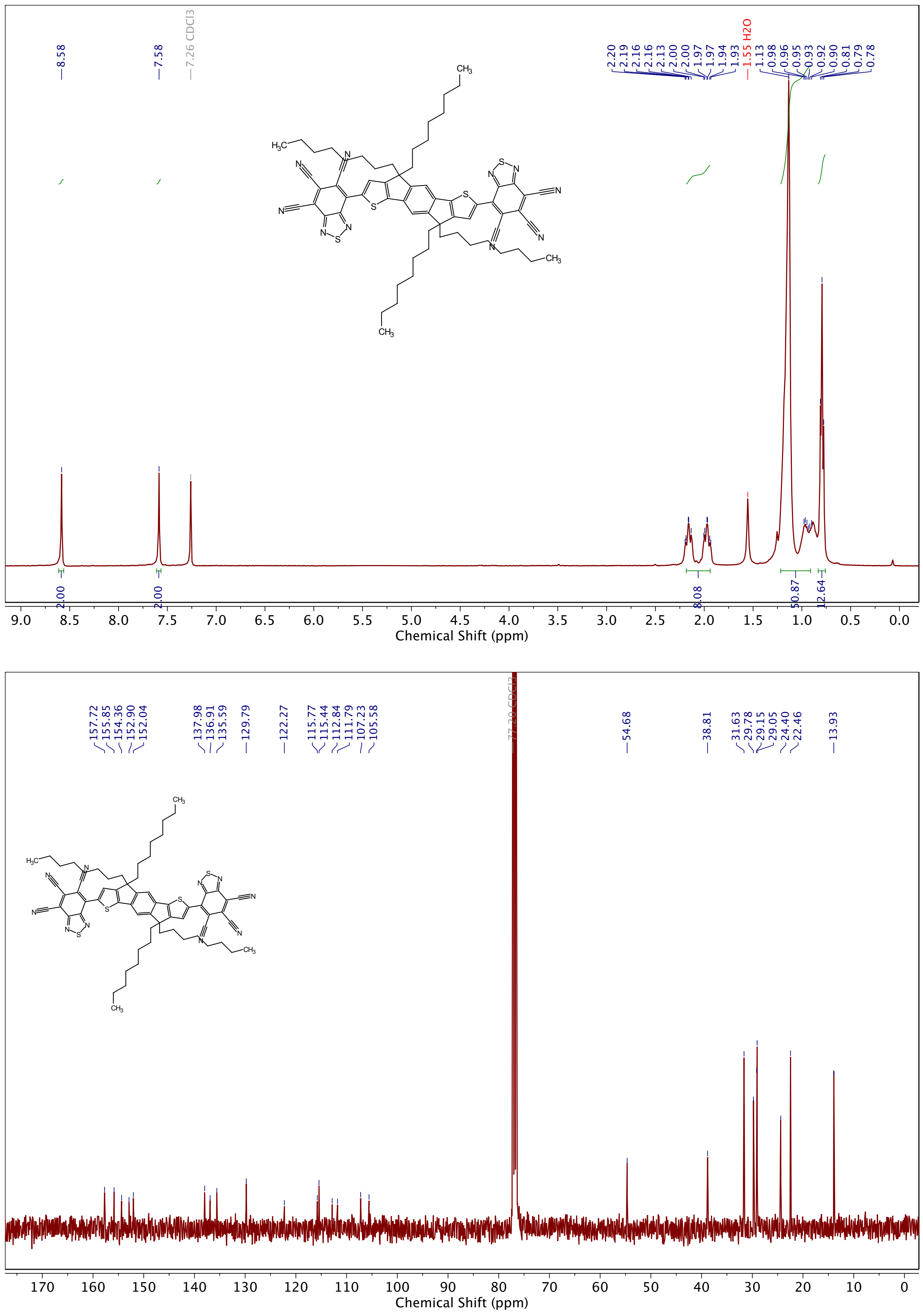

Figure S32. ^{1}H NMR (top) and ^{13}C NMR (bottom) spectra of **TCNBT IDT** in $CDCl_3$.

References

1. Zalesskiy, S. S.; Ananikov, V. P., Pd2(dba)3 as a Precursor of Soluble Metal Complexes and Nanoparticles: Determination of Palladium Active Species for Catalysis and Synthesis. *Organometallics* **2012,** *31* (6), 2302-2309.
2. Bard, A. J.; Faulkner, L. R., *Electrochemical methods : fundamentals and applications*. 2nd ed. ed.; John Wiley: New York ; Chichester, 2001.
3. Koopmans, T., Über die Zuordnung von Wellenfunktionen und Eigenwerten zu den Einzelnen Elektronen Eines Atoms. *Physica* **1934,** *1* (1), 104-113.
4. Baikie, I. D.; Grain, A. C.; Sutherland, J.; Law, J., Dual Mode Kelvin Probe: Featuring Ambient Pressure Photoemission Spectroscopy and Contact Potential Difference. *Energy Procedia* **2014,** *60*, 48-56.
5. Frisch, M. J.; Trucks, G. W.; Schlegel, H. B.; Scuseria, G. E.; Robb, M. A.; Cheeseman, J. R.; Scalmani, G.; Barone, V.; Petersson, G. A.; Nakatsuji, H.; Li, X.; Caricato, M.; Marenich, A. V.; Bloino, J.; Janesko, B. G.; Gomperts, R.; Mennucci, B.; Hratchian, H. P.; Ortiz, J. V.; Izmaylov, A. F.; Sonnenberg, J. L.; Williams; Ding, F.; Lipparini, F.; Egidi, F.; Goings, J.; Peng, B.; Petrone, A.; Henderson, T.; Ranasinghe, D.; Zakrzewski, V. G.; Gao, J.; Rega, N.; Zheng, G.; Liang, W.; Hada, M.; Ehara, M.; Toyota, K.; Fukuda, R.; Hasegawa, J.; Ishida, M.; Nakajima, T.; Honda, Y.; Kitao, O.; Nakai, H.; Vreven, T.; Throssell, K.; Montgomery Jr., J. A.; Peralta, J. E.; Ogliaro, F.; Bearpark, M. J.; Heyd, J. J.; Brothers, E. N.; Kudin, K. N.; Staroverov, V. N.; Keith, T. A.; Kobayashi, R.; Normand, J.; Raghavachari, K.; Rendell, A. P.; Burant, J. C.; Iyengar, S. S.; Tomasi, J.; Cossi, M.; Millam, J. M.; Klene, M.; Adamo, C.; Cammi, R.; Ochterski, J. W.; Martin, R. L.; Morokuma, K.; Farkas, O.; Foresman, J. B.; Fox, D. J. *Gaussian 16 Rev. C.01*, Wallingford, CT, 2016.
6. Becke, A. D., Density-functional thermochemistry. III. The role of exact exchange. *The Journal of Chemical Physics* **1993,** *98* (7), 5648-5652.
7. Petersson, G. A.; Al-Laham, M. A., A complete basis set model chemistry. II. Open-shell systems and the total energies of the first-row atoms. *The Journal of Chemical Physics* **1991,** *94* (9), 6081-6090.
8. Stephens, P. J.; Devlin, F. J.; Chabalowski, C. F.; Frisch, M. J., Ab Initio Calculation of Vibrational Absorption and Circular Dichroism Spectra Using Density Functional Force Fields. *The Journal of Physical Chemistry* **1994,** *98* (45), 11623-11627.
9. Laury, M. L.; Carlson, M. J.; Wilson, A. K., Vibrational frequency scale factors for density functional theory and the polarization consistent basis sets. *Journal of Computational Chemistry* **2012,** *33* (30), 2380-2387.
10. Zhang, W.; Smith, J.; Watkins, S. E.; Gysel, R.; McGehee, M.; Salleo, A.; Kirkpatrick, J.; Ashraf, S.; Anthopoulos, T.; Heeney, M.; McCulloch, I., Indacenodithiophene Semiconducting Polymers for High-Performance, Air-Stable Transistors. *Journal of the American Chemical Society* **2010,** *132* (33), 11437-11439.
